# Ninety-eight semesters of cytochrome P450 enzymes and related topics—What have I taught and learned?

**DOI:** 10.1016/j.jbc.2024.105625

**Published:** 2024-01-05

**Authors:** F. Peter Guengerich

**Affiliations:** Department of Biochemistry, Vanderbilt University School of Medicine, Nashville, Tennessee, USA

**Keywords:** cytochrome P450, enzyme kinetics, enzyme mechanisms, steroid metabolism, electron transfer, oxidation-reduction, toxicology drug metabolism, mentoring

## Abstract

This Reflection article begins with my family background and traces my career through elementary and high school, followed by time at the University of Illinois, Vanderbilt University, the University of Michigan, and then for 98 semesters as a Vanderbilt University faculty member. My research career has dealt with aspects of cytochrome P450 enzymes, and the basic biochemistry has had applications in fields as diverse as drug metabolism, toxicology, medicinal chemistry, pharmacogenetics, biological engineering, and bioremediation. I am grateful for the opportunity to work with the *Journal of Biological Chemistry* not only as an author but also for 34 years as an Editorial Board Member, Associate Editor, Deputy Editor, and interim Editor-in-Chief. Thanks are extended to my family and my mentors, particularly Profs. Harry Broquist and Minor J. Coon, and the more than 170 people who have trained with me. I have never lost the enthusiasm for research that I learned in the summer of 1968 with Harry Broquist, and I have tried to instill this in the many trainees I have worked with. A sentence I use on closing slides is “It’s not just a laboratory—it’s a fraternity.”

I appreciate the opportunity to contribute this Reflection to JBC. By the time this is published, I will be in my 98th semester as a faculty member at Vanderbilt University. Since I started doing research in the summer of 1968 while an undergraduate student, JBC has been held in high regard in the field of biochemistry. I have authored or co-authored at least 135 original papers in JBC to date and also a number of reviews. As detailed later, I spent 18 years on the editorial board and 16 as an associate editor. As I look back on the last 50+ years, I cannot complain (much)—I have had a career I never dreamed of, and I am still happily in business.

## Early life

If you want to understand me, you need to know where I came from. I can trace the Guengerich (then Güngerich) family back to ∼ 1800 in Bavaria (Germany), although there is some rather anecdotal evidence of earlier emigration there from Switzerland. My grandfather, Peter Guengerich, moved from Munich to the United States in 1913, with his wife and six children, including my father, Peter Guengerich Jr. My grandfather followed several of his brothers, each of whom had come to build a better life. He was a common laborer in Germany and had to borrow money for the ship (the “Amerika”) from his relatives. When he arrived in Illinois, among his other relatives, he got a job working for a farmer as a “hired man.” By 1920, he was able to borrow enough money to buy some equipment and began farming himself, share-cropping as a tenant (I will always have a warm spot in my heart for hard-working immigrants).

My father helped with the farm work. He was 8 years old when he arrived and had to learn English. Dad never went to high school, but he eventually went on to purchase his own farm and became successful. He married my mother, Louise (nee Weyhrich—also of German descent), in February 1948, and I entered this world on New Year’s Day in 1949. (So now you know how old I am, but you can pretty easily find that piece of information anyway.)

**Figure 1:**
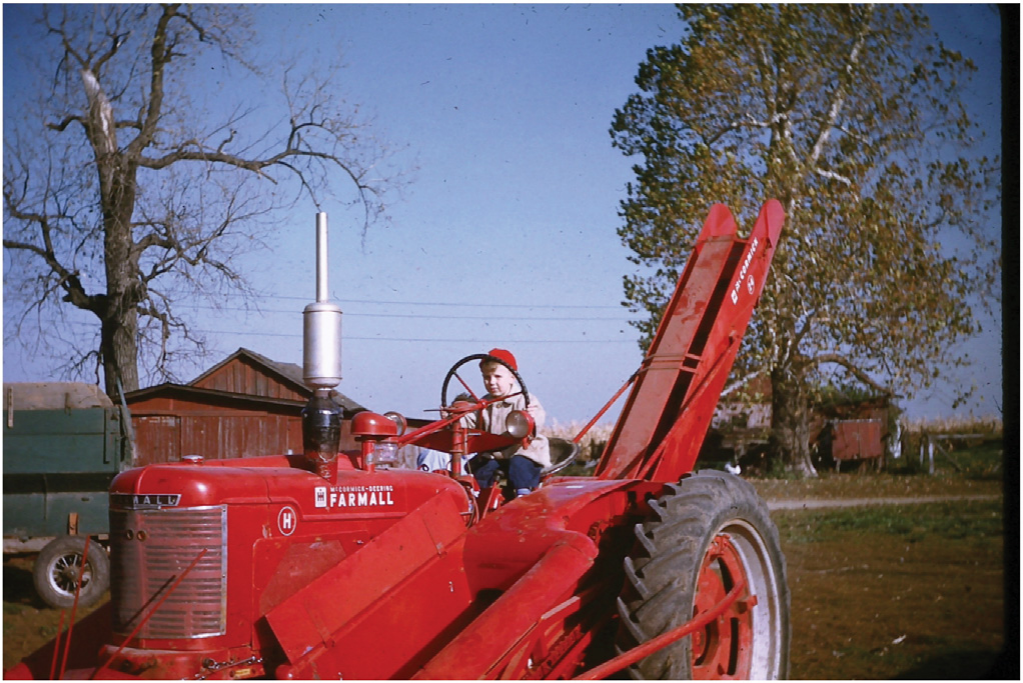
**The author at work driving a corn picker (ca. age 5).** As mentioned, my father had me working on the farm at a young age, but not quite yet—this shot was staged. I did not learn to drive that tractor (Farmall Model H) until the age of 10. (Incidentally, corn pickers have been replaced today by combines.)

My parents were good people, and I have great respect for them. Dad worked very hard all his life. One night when I was 10, Dad told me he thought I was old enough to drive a tractor. He gave me the instruction manual and told me to learn the gearshift pattern because he would show me how to drive it the next day (Fig. 1). By the age of 12, I was also driving the truck (and not just a pickup) (all this was rather common for farm kids, at least then). Driving a car (legal age of 16) was very easy, then, as shifting the gears was much smoother. I helped my father milk the cows each morning and evening. We never had a real vacation, and every Saturday, I was working with him all day, as well as all summer—forget things like soccer practice (actually no one played soccer then anyway). These work habits proved to serve me well later in life; people can criticize me for various things, but I do not think anyone has ever seriously questioned my work ethic. Dad really taught me this. Trust me—it is much easier working in an air-conditioned lab than doing manual labor outside in the cold, heat, dust, and dirt. In those days, tractors did not have nice air-conditioned cabs—you sat out in the elements (Fig. 1).

I was doing well in grade school, and my parents—despite their limited educational backgrounds—encouraged me and even expected me to be getting good grades. This pattern continued into high school. Frankly, I found most of high school pretty boring and was anxious to get it over with. The two subjects that I did enjoy in high school were agriculture and chemistry. With regard to agriculture, I was active in 4-H Club and Future Farmers of America, even rising to the Future Farmers of America Star State Farmer level—top individual that year (1966) in a three-county district (Illinois Section 12). Chemistry had a special allure, though—I was really mesmerized by it, even if I did not understand it all that well then by my standards today. I could tell one story of a personal lab safety accident in our advanced chemistry class in high school, but I would probably get kicked off the Vanderbilt Chemical Safety Committee (my friend Bob Jarchow probably remembers it). I was particularly fascinated by organic chemistry and the ability to make new compounds, although there was not much there in the high school lab yet, even in the advanced course.

I had come to realize that Dad’s farm was too small to be viable in the long-term future and if I wanted to farm myself, I would have to borrow a lot of money to expand. I considered becoming a veterinarian, for a while (although now I know that my animal surgical skills are pretty poor). I entered college (the University of Illinois) in food science/technology—that was a suggestion of one of the high school teachers, and I got a nice scholarship for this (long story, but as part of the visit to the sponsor, I got to meet (the late) Dick Butkus, formerly of the University of Illinois and the legendary middle linebacker of the Chicago Bears football team, and I still have an autographed football from him).

**Figure 2:**
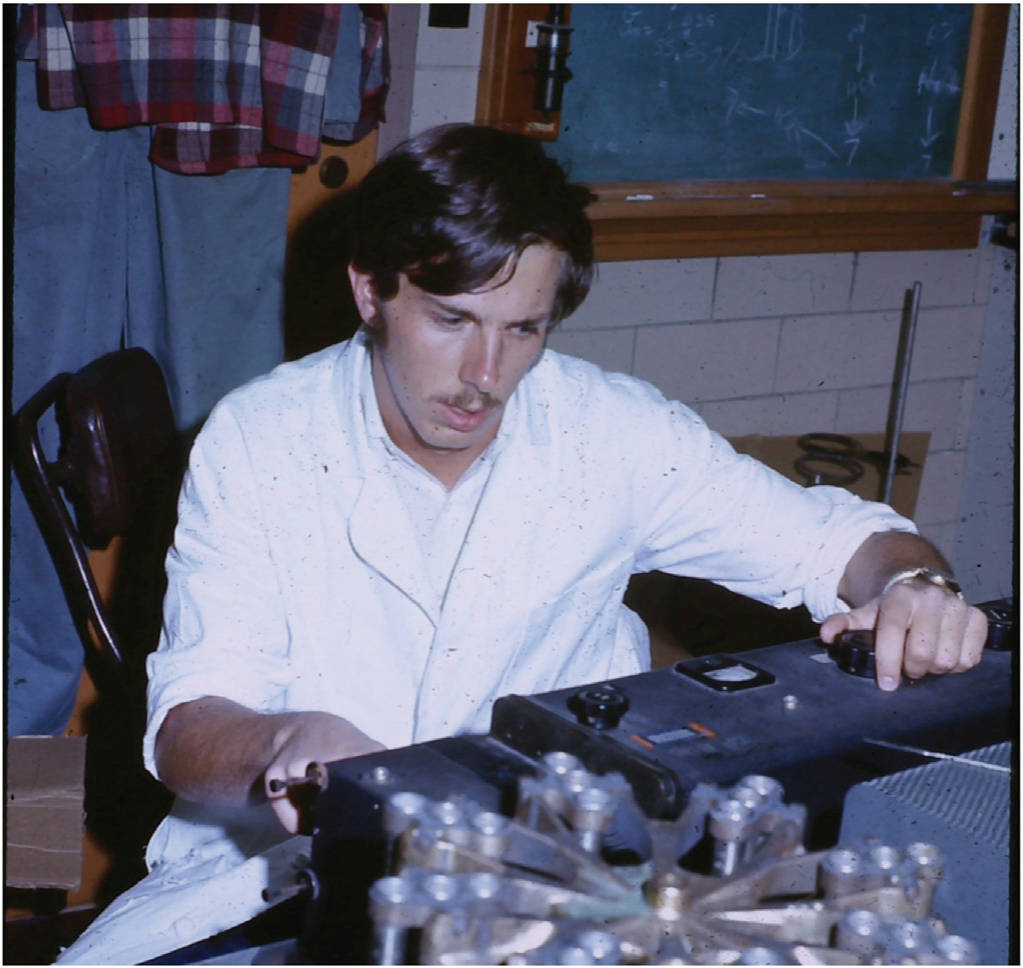
**The author in college (early 1970).** The instrument is a Beckman Model DU spectrophotometer (popular then), with a centrifuge rotor in the foreground.

I was doing well when I started college and got all A grades the first semester (that was harder then, before teaching evaluations started and grade inflation became rampant). I was handling chemistry well, thanks in large part to the good high school preparation. I still appreciate the excellent instruction in chemistry I received as an undergraduate at Illinois. By the middle of my sophomore year, I decided that I would not be happy just getting a degree in food science. I thought about a major in nutrition. My adviser at the time, Prof. Carl Davis, suggested that I spend a summer working in Prof. Harry Broquist’s lab, and I got National Science Foundation support for this. Once I started (1968), I never wanted to do anything else for a career but be a biochemist. During the school year, I was working at a paying job for 20 h a week, with Prof. Charles Graves, in his Physiology lab (Fig. 2). I spent the next summer doing research in Harry Broquist’s lab again. That summer, Prof. Broquist (known as “The Chief”) announced that he was moving to Vanderbilt University. He asked me to consider applying to graduate school there, although he was willing to write me a good letter for anywhere else. Although my undergraduate degree was formally in the College of Agriculture (Agricultural Science), I had 43 h of chemistry (all A’s, more coursework than required for a Chemistry major) and graduated with University Honors (Bronze Tablet, upper 3% of the entire university graduating class).† I only applied to Vanderbilt and Duke and was accepted in both biochemistry graduate programs. I had come to know The Chief well and accepted the offer in Biochemistry at Vanderbilt, although in retrospect Duke probably had a higher profile department at the time.

## Graduate school and postdoctoral studies

I was awarded an individual NIH fellowship for graduate school (F01, equivalent to what is now F31), at the start of entering graduate school (I applied as an undergraduate). The stipend was a whopping $2400 per year then (uncorrected for today’s inflation, of course), but I could live on that. The transition to graduate school at Vanderbilt was relatively easy, in that my coursework in chemistry (esp. organic and analytical) and biochemistry at Illinois had been very solid and I understood much of the background for my project. I was already used to working in a lab most of my spare time, and I was able to pass the preliminary qualifying examination (“prelims”) and present a talk at the American Society for Biochemistry and Molecular Biology (ASBMB) meeting in San Francisco after my first year (that was the first airplane trip I had ever taken). I remember being very nervous for my first real talk outside of Vanderbilt—that may be hard to believe for those of you who know me, in that I am no longer fazed at all by speaking to a large audience (and I have not written out a talk since graduate school).

There was another wrinkle to graduate school in those days. You had to pass a standardized foreign language exam—choice of German, French, or Russian, as I recall. Due to my agrarian background in high school and choices of classes, I had managed to never study a foreign language in my life (my father knew German, of course, but seldom spoke any, and I discovered later that whenever he and my aunts spoke German it was a very thick Bavarian dialect anyway). I took an introductory course (5 classes a week) in German during the first summer I was in graduate school and practiced reading German chemical and medical journals in the library in my spare time—and I passed the exam on the first try. (The requirement was dropped a few years later.)

At the time I started graduate school, I was attracted to the idea of doing a combined M.D.-Ph.D., in order to have more insight into medical research. The usual way of entry is to do the first 2 years of medical school and then the graduate years. I had not taken the medical school exams until my senior undergraduate year, so I started graduate school (at Vanderbilt) and then applied to medical school. I was accepted in medical school (at Vanderbilt) and into their M.D./PhD program. I decided to decline medical school, though—when I saw the med students studying anatomy and physiology, I knew I would hate all the memorization, and I had never really been interested in ever practicing medicine anyway. I suppose I could have been an M.D., but I have never had any regrets about my decision. I think that my research has had some medical relevance anyway (more later).

**Figure 3:**
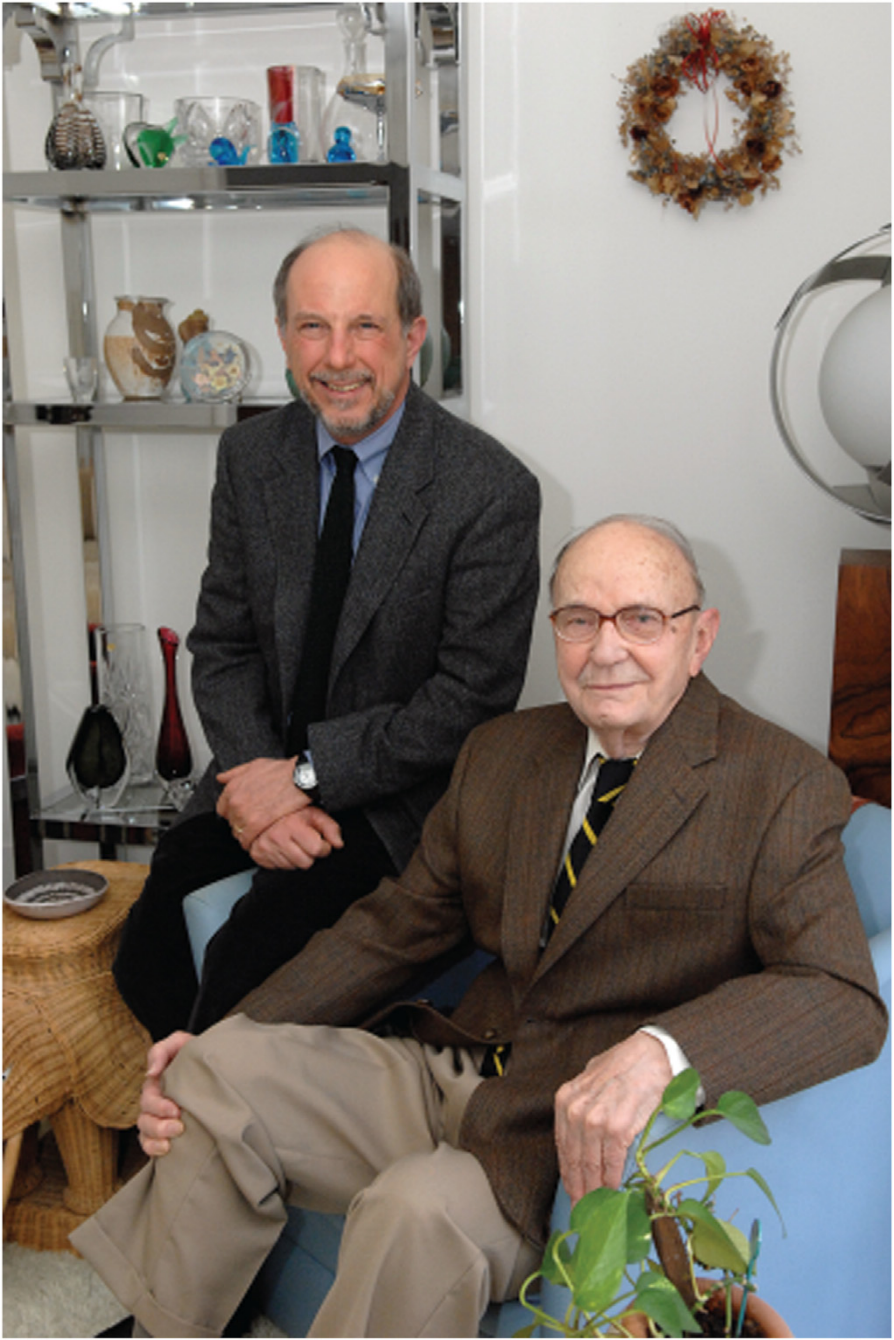
**The author (*left*) with Ph.D. thesis mentor Harry Broquist (*right*).This wasin 2007 on the occasion of receiving an endowed chair at Vanderbilt**.

I primarily studied the biosynthesis of alkaloids in my PhD thesis work (1, 2, 3, 4) (Fig. 3). Today, this area is more popular (“bioorganic chemistry” or “chemical biology”). I will not belabor this work, but I did learn a lot about organic synthesis, chromatography, spectroscopy, *in vitro* assays, natural products, and working with small amounts of chemicals—real microchemistry. These lessons would all help me a great deal in studying drug metabolism later. What I wanted to learn more about, though, was serious enzymology. For postdoctoral work, I received offers from Profs. Esmond Snell (pyridoxal, Berkeley), Donald McCormick (flavins, Cornell), and Minor J. (“Jud”) Coon (cytochrome P450 (P450), Michigan). Jud was the only one who invited me for a paid visit (seems unusual now!) and offered me the highest stipend. My decision to work with Jud Coon was not based on these perks, but in retrospect, it was definitely the right one given how things turned out. I thought there might be practical applications for P450 research someday, especially if I decided to go into industry. I did not expect to keep working on the same enzymes for the rest of my life, but here I am and hence the title of the article.

**Figure 4:**
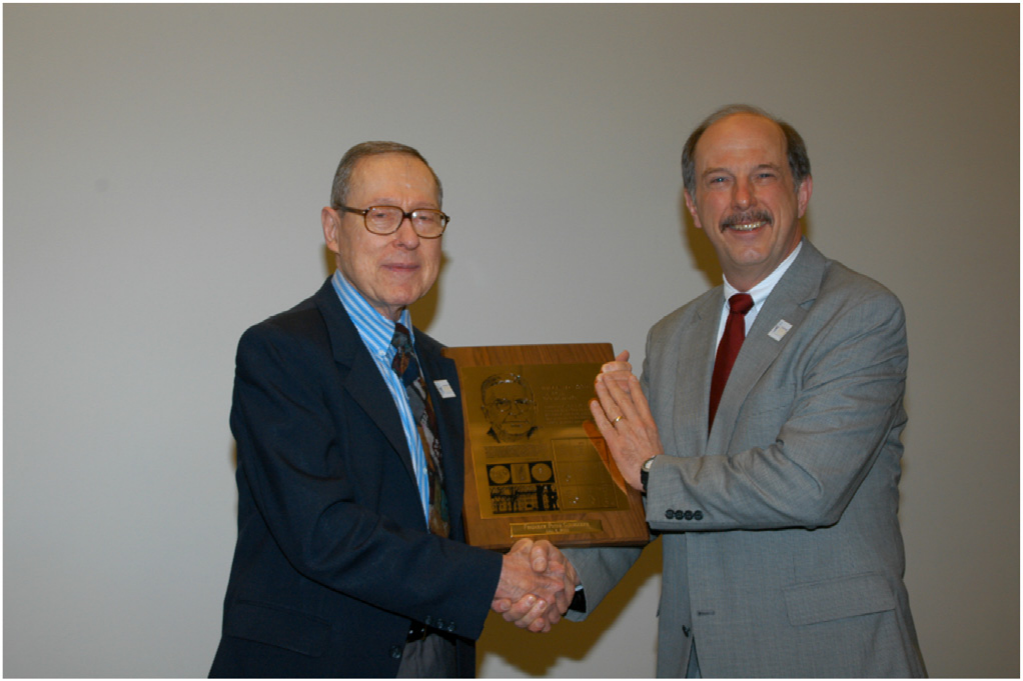
**The author’s postdoctoral mentor Jud Coon (*left*) presenting him (*right*) the William C. Rose Award at the ASBMB meeting in 2005.** (Note: Prof. Rose was Prof. Coon’s mentor, so this award represents three generations of biochemists, all affiliated at some point with the University of Illinois.)

I am very blessed to have had such good mentors, and I have tried hard to be a good mentor myself (more about that later). Harry Broquist really got me excited about biochemistry and showed me that I could be successful. In retrospect, it was Jud Coon who really taught me how to be a professor, although I did not always realize it at the time (Fig. 4).

As an interlude, I met a beautiful red-headed nurse in my last year of graduate school. I told her I was planning to leave for Michigan in 6 months. I soon realized I was in love, and so was she. We were engaged in short order, and then I left for Ann Arbor. Cheryl Powell and I were married in December 1973, shortly after I started as a postdoc, and she moved in. We have been happy together since then, although there were probably times that she considered biochemistry to be a very demanding mistress of mine.

Jud Coon’s lab was first-rate, but the research problems were hard and P450 was already a competitive field. P450 purification work was still in its infancy. I did work on redox titrations, component interactions, and kinetics, particularly with iron-oxygen complexes (5, 6, 7). I should also add that I picked up things from others at the University of Michigan while I was there. I learned from others in our lab how to purify proteins, even though that was not part of my own project. Jud did not do lab work himself (after all, he was the department chair and I appreciate that he was busy), but there were two faculty members who really did work in their labs—Dave Ballou and Vince Massey, the pope of flavins. I learned a lot about practical anaerobic techniques and kinetics from Dave. In a sense, I have probably tried to emulate Vince, who challenged his trainees to outdo him and continued to work in his lab until literally the day before he died. Vince had a mind like a steel trap—I wish I did. He could conjure up detailed information from decades ago.

During the middle of my second year as a postdoc, Harry Broquist asked me if I would be interested in applying for a faculty position back in the Vanderbilt Department of Biochemistry that had become available (because one of the faculty on my thesis committee had not been renewed!). At this point, I was not really sure where I wanted to work. I liked living in Michigan and probably would have taken a good offer in the pharmaceutical or chemical industry if I had gotten one. To be honest, I was intimidated about playing the “grants game” in academia (It is much worse today than it was then, but I have been continuously funded by NIH to date, at least as of now). I went back to Nashville to give a seminar, they offered me a job as an assistant professor, and the rest is history. Things were not as complex as today, with the almost requisite papers in *Nature* or *Cell*, elaborate research proposals, diversity plan write-ups (still legal?), pre-interview Zoom meetings, long visits, chalk talks, and second visits. To be honest, I was not exactly sure what I would start working on when they hired me, but I was supposed to somehow fit into the Center in Environmental Toxicology, then directed by Prof. Robert Neal. I think at least some of the faculty in the department must have been impressed with me as a graduate student, so the earlier decision to do my thesis work with Harry Broquist at Vanderbilt turned out to be pivotal in my career. So, students, take note—people may be watching you, too.

## The late 1970s and life as an assistant professor

My wife Cheryl and I moved back to Nashville in September 1975. This move was the last thing on my mind 2 years earlier when I left Nashville for Ann Arbor as a newly-minted Ph.D. with all my earthly possessions piled in my 1964 Ford. Starting my own lab was a big transition, one of a magnitude I had never experienced previously in my life. They give you the keys to your lab and you suddenly realize that you have to do everything on your own now! Then Prof. Leon Cunningham, Chairman at that time, dropped by my lab and told me they really expected big things of me. When he told me that, my main thought was just how to survive. I think the faculty accepted me as a colleague—most of the department staff seemed to think I was still a grad student, though.

Within about 2 weeks after arriving, I had to present my research program for the NIH site visit for the Toxicology Center (NIEHS P30 grant), directed by Bob Neal. I proposed to study the bioactivation of a series of model and environmental chemicals, namely furans, nitrosamines, pyrrolizidine alkaloids, and vinyl monomers by individual rat and (possibly) human P450s. I honestly had little idea about how I would ever master the human P450s, but I threw that in the proposal for kicks. Ultimately, I did succeed in characterizing the reactions of some of each group of these compounds and the human P450s as well.

Getting started on the faculty level was not easy; I do not think it ever is. The first year was a series of constant challenges. Buying my own equipment was fun—but there was a lot of “sticker shock” when I saw how much everything cost, even then. Finding good postdocs is very hard when you are starting out—why should anyone good want to risk working for you? I did not get my first graduate student (Randy Miller) for 3 years (1978). Hiring research assistants proved to be problematic, but it finally clicked with Marge Mitchell and Pat Mason, and then Martha Martin came in 1979 and stayed for 31 years. I was able to work in the lab a lot myself and published several papers as the sole author (8, 9, 10, 11, 12, 13), purifying rat and rabbit P450s and elucidating their activities toward potentially toxic and carcinogenic compounds (9, 13).

I got the first NIH R01 grant I applied for in 1976 (I never failed to be funded with any kind of grant application at NIH or elsewhere until 1999). We did more work on characterizing rat P450s and then human P450s. In 1978, I was invited to talk at an international P450 meeting in Mainz, Germany, my first ever trip abroad. By 1979, I was a regular member of the NIH Physical Biochemistry Study Section, which was rare for assistant professors in those days. I must say I felt intimidated the first time, going to Washington D. C. to be on a panel with the likes of Bob Simoni, Henry Mahler, Ken Neet, Dan Ziegler, and Bill Jencks. What if they did not think much of my reviews? They seemed to accept me, though, and I learned a lot from the grant reviewing experience in the early years, as well as meeting important people. Jeanne Ketley (in charge of the study section) signed me on after the first meeting, and I went on to spend well over 17 years on NIH study sections and council over the years. It is important for good reviewers to participate, although as time went on, I learned fewer new things and more of it became drudgery.

One October day in 1979, Leon Cunningham strolled into my lab, while I was working, and told me the senior faculty had voted to promote me. I did not know they had even met and was surprised, although at the time I had a lot of papers published (including several in JBC), three of my own NIH grants, an NIH Research Career Development Award grant, membership on an NIH study section, and an invited lecture at an international meeting.

## The associate professor years and the early 1980s

The lab was growing now in the 1980s and I began traveling a lot (maybe too much) for speaking engagements. I got to know many people in Europe and Japan. Two trips were particularly memorable—one to Bratislava (in what was then Czechoslovakia, now Slovakia) in 1980 and one to East Berlin in 1985. These countries were behind the “Iron Curtain” at the time and showed me what communism was really like. These experiences, and conversations over the years with two of my former faculty colleague and friends who had escaped what was Czechoslovakia (Lubomir Hnilica and Frank Chytil) (14), have had an impact on my political and social views ever since.

**Figure 5:**
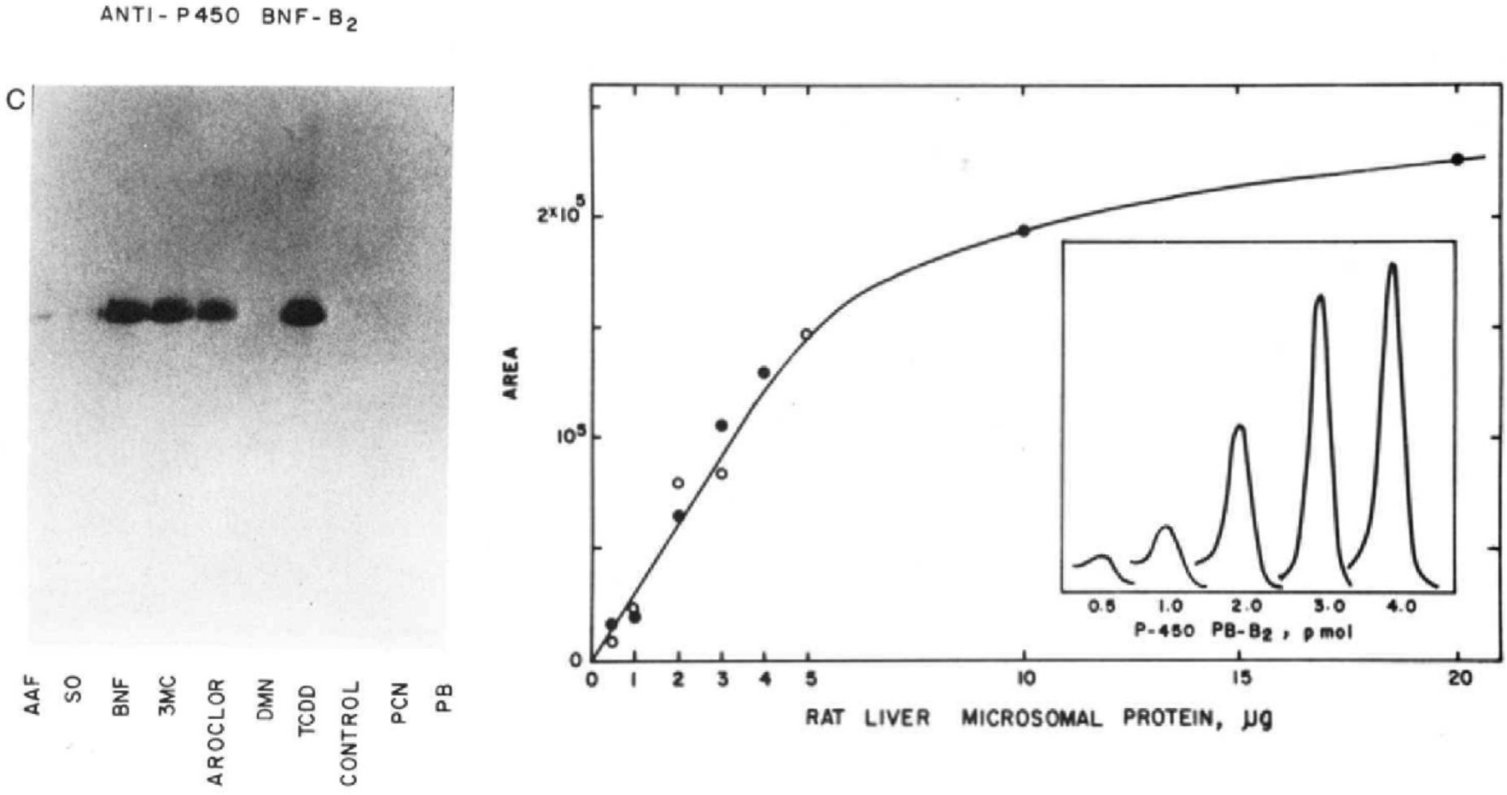
**Immunoblotting in 1981 to 1982** (16)**.** The *left side* shows an electrophoretogram (7.5% acrylamide) of samples of liver microsomes prepared from male rats treated with 2-acetylaminofluorene (AAF), *trans*-stilbene oxide, (SO), β-naphthoflavone (BNF), 3-methylcholanthrene (3-MC), Aroclor 1254 (AROCLOR), *N*,*N*-dimethylnitrosamine (DMN), 2,3,7,8-tetrachlorodibenzo-*p*-dioxin (TCDD), corn oil only (CONTROL), pregenenolone-16α-carbonitrile (PCN), or phenobarbital (PB) and stained using what would today be termed rabbit anti-rat P450 1A1 (then termed BNF-B_2_). The panel on the *right side* shows the integrals of scans of the peaks (inset) detected by rabbit anti-rat P450 2B1 (then PB-B_2_) in liver microsomes of male rats treated with phenobarbital to induce the enzyme. Note the linearity of the response (*open* and *closed circles* are replicates) up to 5 μg protein. In this early work, horseradish peroxidase and benzidine were used to stain the immunoreactive proteins.

We published a major paper on characterization of 8 rat liver P450s in 1982 (15), as well as what I believe is the first paper on quantitative immuno (“Western”) blotting (16), which was a great boon to answering some questions about P450 regulation (15, 17, 18). There is a story behind this. We did not invent immunoblotting, but we were able to get the system of Towbin *et al.* (19) going with P450s and microsomes in our lab (20). Our first system was crude, with two steel plates bolted to electrodes, sandwiching the gel/nitrocellulose paper assembly (with grocery store sponges being held together with rubber bands) and inserted in an open beaker (sounds dangerous now). The intensity of the peroxidase-benzidine bands seemed concentration-dependent. I found someone in our Department of Medicine who had a scanner for TLC plates (there were few computers and imagers around then) and discovered that we could scan the blots if we masked lanes individually (16). Later, blotting would be an issue in the entire field with short displayed zones, lack of controls, etc. (see JBC Instructions to Authors), but in the first work, we showed all of our complete gels and calibration plots (15, 16, 18) (Fig. 5)‡.

We were also doing interesting work on the oxidation of vinyl halides and dihaloalkanes, including some with glutathione transferases and epoxide hydrolase (22, 23, 24). We also began purifying P450s from human liver. This was a difficult endeavor, in terms of obtaining liver samples and doing the purification (25, 26). The outcome of that work has been reviewed already in a JBC Classic (27). At the outset, we had no idea how many P450s we humans have or how many would be important in the metabolism of drugs and carcinogens. Our early success in this field can be credited to a few postdocs, most notably Linda Distlerath and Tsutomu Shimada (28, 29, 30, 31, 32, 33, 34), each of whom went on to a great career.

In 1984, I gave a seminar at St Mary’s Medical School in London at the invitation of Prof. Jeffrey Idle, following the Microsomes and Drug Oxidations meeting in Brighton. Jeff told me I might be interested in characterizing the human P450(s) involved in the oxidation of nifedipine because Prof. Douwe Breimer, at Leiden, had clinical evidence for a polymorphism. I said something brilliant like “What’s nifedipine?” (it was a major antihypertensive drug at the time and one of the 10 best-selling drugs, as I now recall). He gave me some drug and the oxidized pyridine product, which I took home (we used to carry a number of things on planes without mentioning them, but I won’t go into that). I came back and asked the members of my group if anyone wanted to start the project but got no taker. So I set up an HPLC separation for the assay myself; then Martha Martin and I started running human liver microsomes through columns. The fractions each had to have detergent removed and then assays were run and analyzed using HPLC. At that time, we had no HPLC auto-injectors, and I was running 40 assays myself some days, with manual injection—I had the isocratic runs down to 3 min each then (also, the substrate nifedipine is light sensitive and all assays have to be done in amber glass). Ultimately, we purified P450_NF_ (NF for nifedipine, now known as P450 3A4 or CYP3A4), which is involved in the metabolism of half the drugs on the market (35, 36, 37, 38). At the time, we had no idea how big the impact of this P450_NF_ work would be. Within 3 years, we went from setting up an assay to characterizing the enzyme to isolating a cDNA with the help of Philippe Beaune on sabbatical from Paris (39). Today, we know that P450s are involved in the metabolism of ∼ ¾ of (small-molecule) drugs and most of that by 5-6 enzymes: P450s 1A2, 2C8, 2C9, 2C19, 2D6, and 3A4 (37, 38, 40). All but 2C19 were purified from liver tissue in our lab (27, 29, 31, 35).

I am not sure that all of our current students and postdocs appreciate what the field of biochemistry was like then. Enzyme purification from tissues cannot be done with affinity tags, of course, and devising purification schemes was difficult—especially the purification of intrinsic membrane proteins like P450s that requires the inclusion of a detergent. There was no means of obtaining DNA sequences until the early 1980s, and when we finally did, we had to run our own sequencing gels (39, 41, 42). Cloning full-length cDNAs was a monumental task, and much of your success was at the mercy of how good the commercial reagents really were. Another problem, before heterologous expression, was deciding if a protein was really pure by electrophoresis alone. Conversely, it was often difficult to know if proteins that had eluted in separate chromatographic regions were related to the intended target or not (43).

Analytical chemistry was laborious then. As a graduate student, I often ran a single radiolabeled sample through a Dowex ion-exchange column overnight with a gradient, collecting the eluent in test tubes with a fraction collector, took aliquots of each for liquid scintillation counting (before Pipetteman devices), and then plotted the radioactivity (cpm) versus fraction number on graph paper (3). I even had to pour my own TLC plates. I acquired my first HPLC in 1977. As a graduate student, getting even any GC-MS work done was difficult, as there were only two mass spectrometers on campus (in Chemistry and Clinical Pharmacology). On-line LC-MS did not come until ∼ 1990, thanks to Prof. John Fenn. Today, people in my lab routinely run many samples through ultra-performance liquid chromatography systems every day with auto-injection and short run times, and we have LC instruments connected to a radio-flow counter and a mass spectrometer in our own lab.

## Developments in academic life—The 1980s

Let me digress and touch on some other parallel developments. In 1980, Bob Neal decided to leave Vanderbilt to become President of the Chemical Industry Institute of Toxicology in North Carolina, an excellent program (that unfortunately no longer exists). For lack of a better alternative, I suppose, I was approached by Leon Cunningham and agreed to be Interim Director of the Vanderbilt Center in Environmental Toxicology. Vanderbilt made an offer to my late friend Prof. Don Reed, from Oregon State University, to replace Bob Neal but I do not think it was very substantial and he had family issues about moving anyway. I then became Director in 1981 although I did this with some reluctance, but I was to continue for another 30 years. My heart was really in doing research, working with young people, and teaching—not doing administrative work. I had begun to address issues in the chemistry of P450 catalysis, along with Prof. Tim Macdonald, who at the time was in our Department of Chemistry (44, 45, 46, 47, 48). So, then, why did I agree to direct the Center? At the time, the research infrastructure at Vanderbilt was not that great. We had many good scientists but also constant problems keeping up with needs for state-of-the-art equipment. We were sorely lacking in expertise in molecular biology. I saw this as an opportunity to help acquire some of these resources. I had also come to appreciate that too much of toxicology was simply descriptive and that more biochemistry could help answer many of the important questions. I liked to think I was an organized person who could handle this job along with my research—much of which I was still doing myself.

I turned over most of the members of the Toxicology Center, adding new faculty already here. I appreciate the help I received from Profs. Tom Harris and Lubomir Hnilica. We had an NIH site visit for the Center grant in 1982, and I thought I knew a lot from the couple of years of my time on an NIH study section. The site visit team did not seem to think I did—and I did not—but they did give us a 2-years extension anyway. This was probably fair, even if not appreciated at the time. I was determined that we would not have another bad site visit, and we did not.

One day in June 1982, I was talking to Leon Cunningham (still Chairman) in his office, and he told me the senior faculty had voted to promote me to (full) Professor. So in mid-1983, I became a full professor at Vanderbilt at the ripe old age of 34. Not bad, I suppose (I would not get another promotion (to an endowed chair) for another 24 years, though). I had been offered a full professorship, plus a 50% increase in salary, at Texas A & M University 2 years earlier. I am glad I made the decision to stay at Vanderbilt, despite some recurring issues—one of which was lab space. Life here used to be very crowded, but that has finally been solved (at least for now).

Life was good through most of the 1980s. I was a tenured full professor at a very good university. My wife Cheryl and I now had three wonderful children. My parents were still healthy and doing well. I was traveling a lot, especially to Europe and Japan. However, my dad suffered a major stroke in late 1987 and that was a very serious blow (he lived 4-1/2 more years). On a positive note, Cheryl and I decided that we would finally take a real vacation (*i.e.*, not just visiting the relatives) with our children to Yellowstone National Park in 1988. We enjoyed this very much and went on to visit many national parks in the American West for another decade or more. The children were starting to leave for college, though, beginning with Phillip in 1995. We did not get to take a major vacation with all three of our children between the years 1997 and 2020.

The Center in Molecular Toxicology (name changed) was thriving and several new faculty moved here and fit in well. We did very well in the NIEHS site visits in 1984, 1988, 1993, 1998, and 2003, and the companion training grant (T32) kept getting renewed too. But there were storm clouds on the horizon, following changes in the leadership at NIEHS.

Our lab had learned to do recombinant DNA technology in the 1980s, thanks in large part to Prof. Stephen Lloyd, who really brought the technology here and shared it with us (39, 41, 42, 49). My postdoc Dianne Umbenhauer was very courageous in this, in that I had no experience in the area then and really did not know the difference between a Southern and a Northern gel (I guess few know either of these now, anyway)—I remember reading the Maniatis book just to keep up. Ultimately, we were able to express (many) human P450s in *Escherichia coli* (50, 51, 52, 53).

I was still doing a lot of traveling to scientific meetings etc. I went on completely round-the-world trips in 1989, 1992, and (twice) in 1994 (*i.e.*, US to Asia to Europe to US or the opposite direction, stopping for meetings and invited lectures at multiple places). Mike Waterman, an old friend from the P450 world, arrived from Dallas as Department Chair in 1992 (and continued in that role until 2010). We had active collaborations with many faculty at Vanderbilt not only in Biochemistry but also in Chemistry and Clinical Pharmacology, including Profs. Mike Waterman, Tom Harris, Carmelo Rizzo, Larry Marnett, Richard Armstrong, John Oates, Jorge Capdevila, Alistair Wood, Allan Brash, Richard Kim, and Grant Wilkinson. Incidentally, my colleague Stanley Cohen—of our department—received the Nobel Prize in 1986 (Medicine or Physiology).

Because of our success with P450s and the characterization of their roles in drug metabolism, I developed many contacts in the pharmaceutical industry, especially in the areas of drug metabolism/pharmacokinetics and toxicology (54). In 1986, I joined Merck’s Advisory Board in their Department of Safety Assessment. Later, I had a similar role at Schering-Plough (now part of Merck). I should also credit my friend Anthony Lu, long at Merck, for teaching me much about the pharmaceutical industry. I have appreciated seeing the practical sides of the science I was involved in and its relevance. For instance, our work with terfenadine and P450 3A4 (55) helped explain some serious drug–drug interactions including deaths, and the U.S. Food and Drug Administration (FDA) has changed considerably as the science progressed. A large number of the graduate students and postdocs who trained in my lab have gone on to productive careers in the pharmaceutical and biotech industries, and many of them have exerted major impact in their field. I take issue with some of my faculty colleagues who only want their students to attain faculty positions in research-intensive universities and consider industry as being undesirable. Frankly, I want first-rate scientists discovering the medicines I need to use.

## The 1990s

The Center in Molecular Toxicology continued to develop, with the addition of Larry Marnett, Richard Armstrong, Mike Waterman, and others (56). We had a very stimulating visiting seminar program every year. In our lab, we were phenotyping human P450s for their catalytic activities (32, 33, 34). Things had really picked up when we were finally able to express P450s in *E. coli*, thanks in particular to postdocs Liz Gillam and Punam Sandhu (51, 57, 58). Mass spectrometry was getting better, as we moved from fast-atom bombardment to electrospray LC-MS (59, 60, 61, 62). We were also able to establish convincingly that P450s could catalyze oxidations of low redox potential substrates, for example, amines and strained hydrocarbons, *via* (proton-coupled) single electron transfer (63, 64). In 1994, our studies with DNA adducts had moved to the point where we started doing pre-steady-state kinetic analysis of DNA polymerase reactions, using our new rapid quench apparatus that my student Laura (Lowe) Furge set up (65).

One real boon in this period was the R35 grant (Outstanding Investigator Award, OIG) I received from the National Cancer Institute (NCI) in 1987. This allowed me to pool my existing three NIH grants into a single larger, comprehensive one, with minimal details, and it ran for 7 years. I successfully renewed this for another 7 years in 1992, so overall I enjoyed 14 years of pretty flexible funding—from 1987 to 2001. I had some very talented students and postdocs during the period, and in retrospect, it was one of the most productive periods in my career (mostly during my 40s). However, in its pseudo-wisdom, the NCI decided it did not like the OIG program and ended it, and I had to revert to applying for individual R01 awards with specific aims, developed in excruciating detail. Despite the outstanding productivity we had, my R01 P450 proposal was not funded in 1999. This was the first NIH grant application of any kind I had written that was not funded, and I realized that I too was mortal. Unfortunately, it would not be the last failure in grant applications. The NIH was changing.

## The 2000s

Through reapplications at NIH, I did get a P450 grant from NCI again by the end of 2000 and had two other National Institute of Environmental Health Sciences (NIEHS) grants. We were rolling, and in 2002, I was able to finally buy my own stopped-flow spectrophotometer, a beautiful OLIS RSM-1000 machine that we still have and that I can still run myself (66, 67). One of our goals had been to obtain X-ray crystal structures of a DNA polymerase caught in the act of making a misinsertion mistake opposite a DNA adduct. We had a number of false starts, mainly starting with a bad choice of a polymerase (HIV reverse transcriptase, which had been a good model in the kinetic work (68)). Dr. Wei Yang (NIH) achieved this before we could, using *Sulfolobus solfataricus* Dpo4 (69). In collaboration with my departmental colleague Prof. Martin Egli, we seized on Dpo4 and published a crystal structure of it making several mistakes in copying past 1,*N*^2^-ethenoguanine (70). In that paper (which made JBC cover art), we also developed a simple but impactful mass spectrometry approach for the analysis of oligonucleotide products of DNA polymerase extensions, quantifying both misincorporation events and frameshifts. I thought it was a very nice piece of work myself.

August and September of 2001 were very memorable. I received the Sutherland Prize, the highest award for research on the Vanderbilt campus. This is not easy to win, as I knew from my prior experience on the Graduate Faculty Council, who made the selection. The definition of what is outstanding research and scholarship really varies among the faculty in diverse fields such as English, Economics, Humanities, and Natural Sciences. Two weeks later, I left for the International Conference on Cytochrome P450 (ICCP450) in France. I learned of the shocking terrorist attacks in the United States on 11 September after I landed in Charles De Gaulle airport in Paris. I had another meeting in Copenhagen the following week, and after that, the flight schedules home had normalized again.

In 2002, I received a Docteur Honoris Causa (honorary doctorate) from the University of Paris (V), because of the efforts of my friend Philippe Beaune and others. The ceremony was held in the Sorbonne in Paris, in French (which I understood very little, of course—see earlier text about my limited exposure in foreign languages). That meant a lot to me, and I have the large certificate framed in my office. I had come a long way from a farm in Central Illinois to the Sorbonne.

In 2005, my P450 grant was renewed by the NCI with a great score and given MERIT status, that is, extended to 10 years instead of 5. At the end of the 10 years, the NCI (again in their infinite wisdom) decided that they were no longer interested in chemical carcinogenesis and kicked my grant over to NIGMS (NIH General Medical Institute).

The year 2008 was a rough one. This was the first time I visited China (People’s Republic), which was interesting. My mother was in failing health, though, and I got the news of her death by phone in Shanghai. Fortunately, it was the day I was leaving. My wife, Cheryl, was diagnosed with breast cancer and had surgery but fortunately has been cancer-free since. The stock market tanked at the end of the year. A Vanderbilt colleague and collaborator, Prof. Jason Morrow, head of Clinical Pharmacology, died by suicide—this was a real shock. We had planned for him to develop a translational program for the Center in Molecular Toxicology NIH P30 grant renewal that was now requisite. The NIEHS, in their own sense of wisdom, was no longer doing site visits. We did not make the cut that time, and it took another year to win back NIEHS funding for the Center, but we then had the best score of the applications. I now really saw the writing on the wall at NIH with regard to funding priorities.

## The JBC

My first appointment to the JBC Editorial Board was in 1984. I remember that on the very first day of my term, I got three manuscripts from different associate editors (they came by mail then, in green folders, so you could readily spot them). I continued to serve four consecutive terms on the JBC Editorial Board, for a total of 18 years. I recall that one year, I reviewed a total of 56 papers for JBC. I had developed a reputation for returning reviews promptly. I thought I was just doing my job but apparently I was noticed for my efficiency.

One day in January 2006, I came back to my office from our laboratory group meeting and there was an e-mail message from Herb Tabor, the JBC Editor-in-Chief, asking me to serve as an Associate Editor (AE). I called some of the AEs I knew (Bill Smith, David Russell, Dick Hanson) and asked how much time this took. They all said about an hour a day—*every* day—*if* you were efficient. Along with my work ethic (*vide supra*), I have long had a general reputation for efficiency. So I agreed to the proposition from Herb.

Why would anyone want to be an editor? I have heard this question from colleagues. A simple answer is that you are paying the system back (or forward), being a part of it. But it is more than that. I think it was the late AE John Exton who told me this—you often come back to your lab from committee meetings and ask yourself if your presence there had even mattered—usually not (I concur). However, when you are an editor, you make real decisions and they are important—and they stick (usually). These, along with grant reviews, can help shape an entire research field.

JBC AEs must have their own manuscripts reviewed (by other AEs). When I became an AE, I decided that we would not submit any manuscripts to JBC that I was not convinced would be accepted. To be honest, in earlier days, there were some manuscripts we pitched on the hope that they would just make it over the fence, so to speak. However, I thought it would be embarrassing to have my own paper declined by a fellow AE. Also, if a marginal paper were accepted, readers might think I was getting favors. I can only recall one submitted paper that (after a long process) did not get accepted at JBC because we could not get enough fluorescent signal out of one experiment. Before I became an AE, we used to publish a lot in *Biochemistry*. It was a fine journal then (and my colleague Richard Armstrong became the Editor), but upon becoming a JBC AE, I thought I should send our best papers to the JBC.

**Figure 6:**
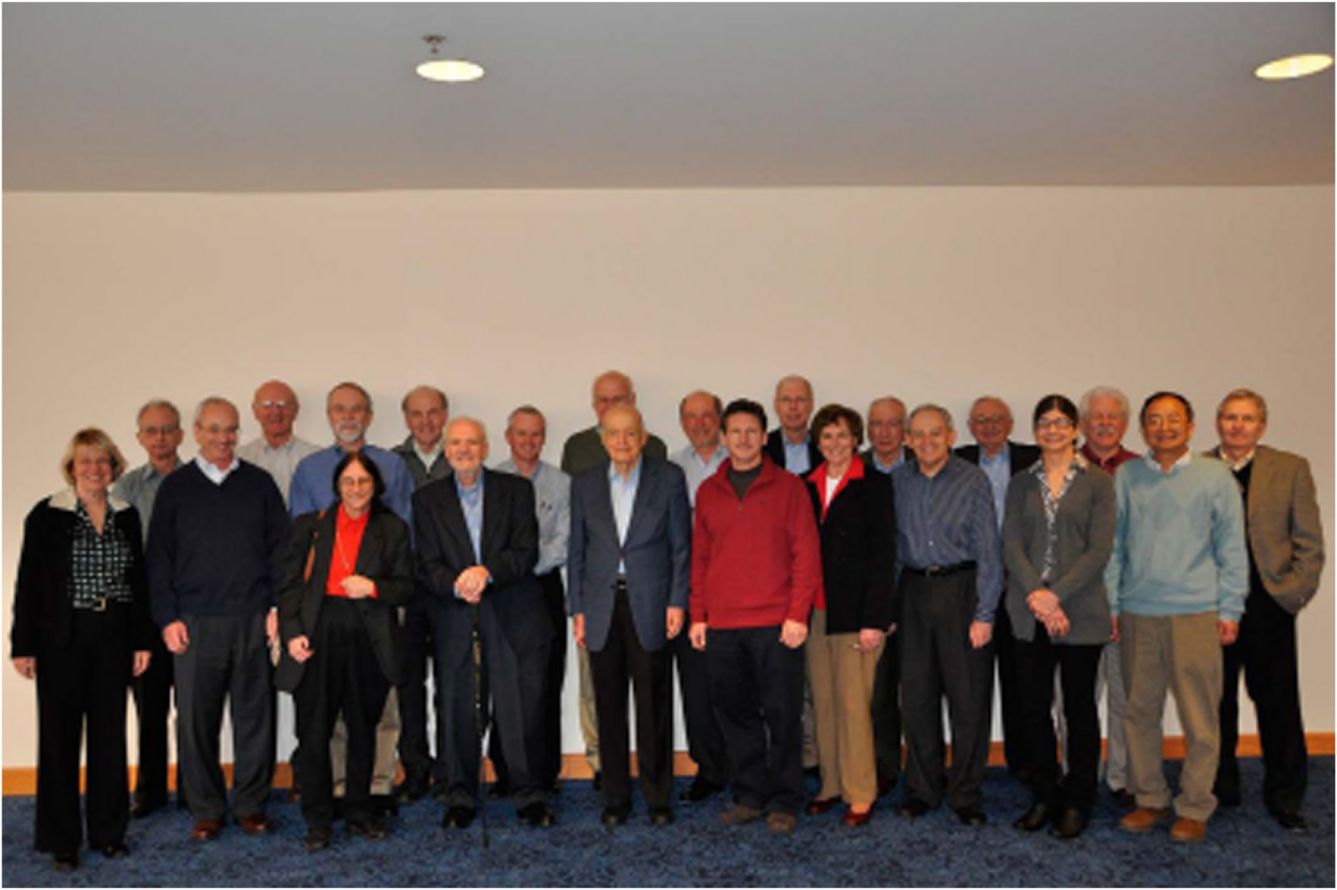
**JBC associate editors at their meeting in San Francisco in February 2010.***Back* (*left to right*): Vince Hascall, Jerry Lingrel, Jim Stull, Ken Neet, David Russell, Chuck Samuel, the author, Jim Siedow, John Exton, Bob Lehman, Bob Simoni, and George Carman. *Front* (*left to right*): Norma Allewell, Bill Smith, Linda Spermulli, Tom Vanaman, Herb Tabor, Dale Benos, Judy Bond, Joel Gottesfeld, Martha Fedor, and Xiao-Fan Wang. Unfortunately, Drs Neet, Siedow, Exton, Simoni, Tabor, and Benos are no longer with us (deceased). (Note: figure previously published in JBC—W.L. Smith Reflection, 2019, volume 294, issue 6, pp. 1779–1793.)

**Figure 7:**
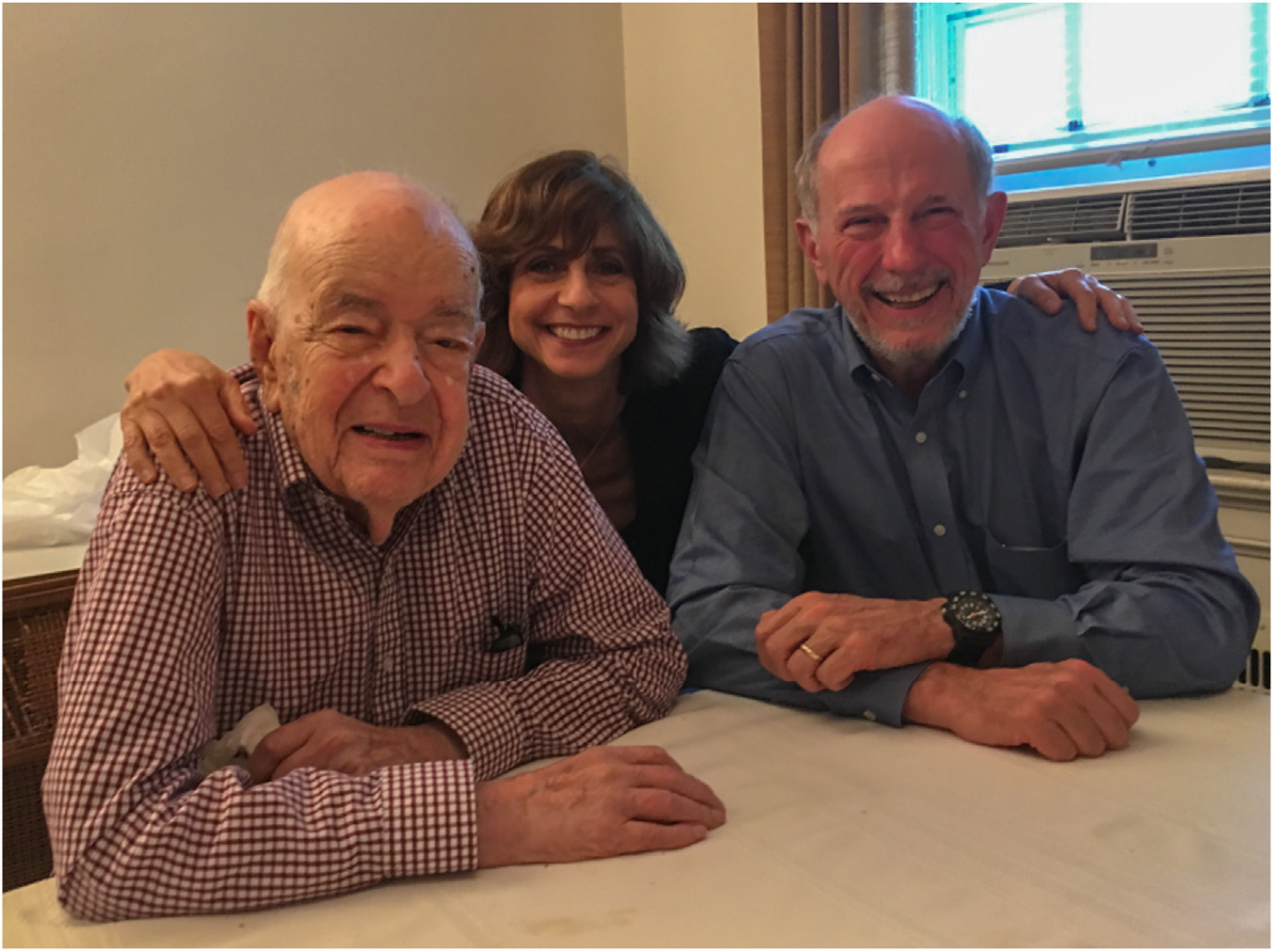
**Lunch at Herb Tabor’s home on the NIH campus (September 2019).***Left to right*: Herb Tabor, Nancy Rodnan (then ASBMB Director of Publications), and the author.

I loved being an AE in those days. Things were different then. JBC paid my secretary, Kathy Trisler, half-time as my assistant, which was typical (it took that much work—one year I handled >600 manuscripts). The AEs met for a 1-1/2 day meeting 3 times a year (Fig. 6). My experience with Herb Tabor was great—this man was just so dedicated to science and to JBC. I have written about him elsewhere (71, 72), but I count working with him as one of the real treasures of my career (Fig. 7). He seemed ageless (but he did finally succumb at the age of 101). But Herb decided to retire as Editor-in-Chief in 2010.

In 2013, I was named deputy editor. There was not much expected at the time, but that changed quickly in 2015 when the then Editor-in-Chief’s appointment was not renewed. I was leaving for the ICCP450 meeting in Tokyo one June morning and checked my iPhone when I got to the Nashville airport to find that I had a message from Barbara Gordon (ASBMB executive director) telling me that Steve McKnight (ASBMB president) wanted to talk to me. I had a sinking feeling I knew why, and I was right. I did talk to him before I left, and now I was Interim Editor-in-Chief. This continued for a year before a new Editor-in-Chief was appointed, and I went back to being deputy editor.

Since 2015, until my term finally ended in 2022, I had to oversee problematic cases in data integrity. Fortunately, I was helped most of the time by Dr Kaoru Sakabe in the JBC front office. Ultimately, I would have to decide what to do about every case, but Kaoru would explain each analysis and we tried to keep our decisions consistent. We probably had a case to deal with almost every day, innocuous or actually a problem, which I handled along with my responsibility of assigning newly submitted manuscripts to AEs 3 to 4 days every week, plus my own manuscript assignments.

Scientific publishing has changed dramatically since 2006, and I have to say I enjoyed the early days more, even though I agree that there have been notable improvements. It has changed not just at JBC but through the whole enterprise—obsession with Journal Impact Factors, Open Access, economic issues, pre-print journals, promoting papers with tweets, data integrity issues, … Much has been written on this topic, for example (73); I have my own thoughts on the matter, more extensive than I have room to write about here. I strongly believe in society-based journals and continue to publish in them, but I am concerned about some general trends, particularly among many of my faculty colleagues. I truly believe in peer review and am not enthusiastic about nonreviewed preprints posing as papers, for legal as well as scientific reasons.

I am glad I had the opportunity to serve JBC for 34 years in various capacities. Having said that, I do not miss the demanding need to assign manuscripts every morning and afternoon. I miss some of my JBC colleagues, especially Herb Tabor, Nancy Rodnan, and Kaoru Sakabe (Fig. 7). Even after publishing >135 papers, I still get excited about getting new manuscripts accepted in the JBC (67, 74).

## 2010 to 2020

Mike Waterman retired as Chair of our department in 2010. Dean Jeffrey Balser asked me to serve as Interim Chair, which I wound up doing for 2 years. I cannot say I was crazy about doing it, given my love of doing research and my other commitments (*e.g.*, JBC).

I found that I could manage the department in about an hour on most days. At that time, the department handled everything (finances, ordering, student issues, etc.) with about six staff members, including the department manager, Marlene Jayne. She was very efficient and had been there forever. I had been the first PhD student to graduate after she started in our department, and she always liked me (she told personnel to be nice to the grad students, because someday they might wind up working for one of them like she did). All of our faculty were funded, our department had more NIH funding than any other U.S. biochemistry department, and there were relatively few major problems. Looking back, the administration did not provide much in the way of resources, though. This had always been the case with Toxicology, too. I probably wasted too much of my time trying to get an NIEHS SuperFund grant and to initiate a drug safety program at Vanderbilt.

In 2011, I announced my desire to step down as Director of the Center in Molecular Toxicology and the associated NIH P30 (core center support) and T32 (training) grants. For some time, things had been changing at NIEHS, with their emphasis on outreach programs and translational programs at the expense of basic science. Michael Aschner and then Dan Liebler later headed these programs but eventually the P30 grant ran its course by 2015. The training grant is still funded (after 49 years), and I had to get involved again (as director) after Aaron Bowman left in 2017, but I am phasing this out (again) and turning the keys over to Fiona Harrison for the future. I had applied to be Chair of Biochemistry in 2011, on the advice of several colleagues while I had been Interim Chair, but I found that management had no interest in me doing that—another lesson learned.

**Figure 8:**
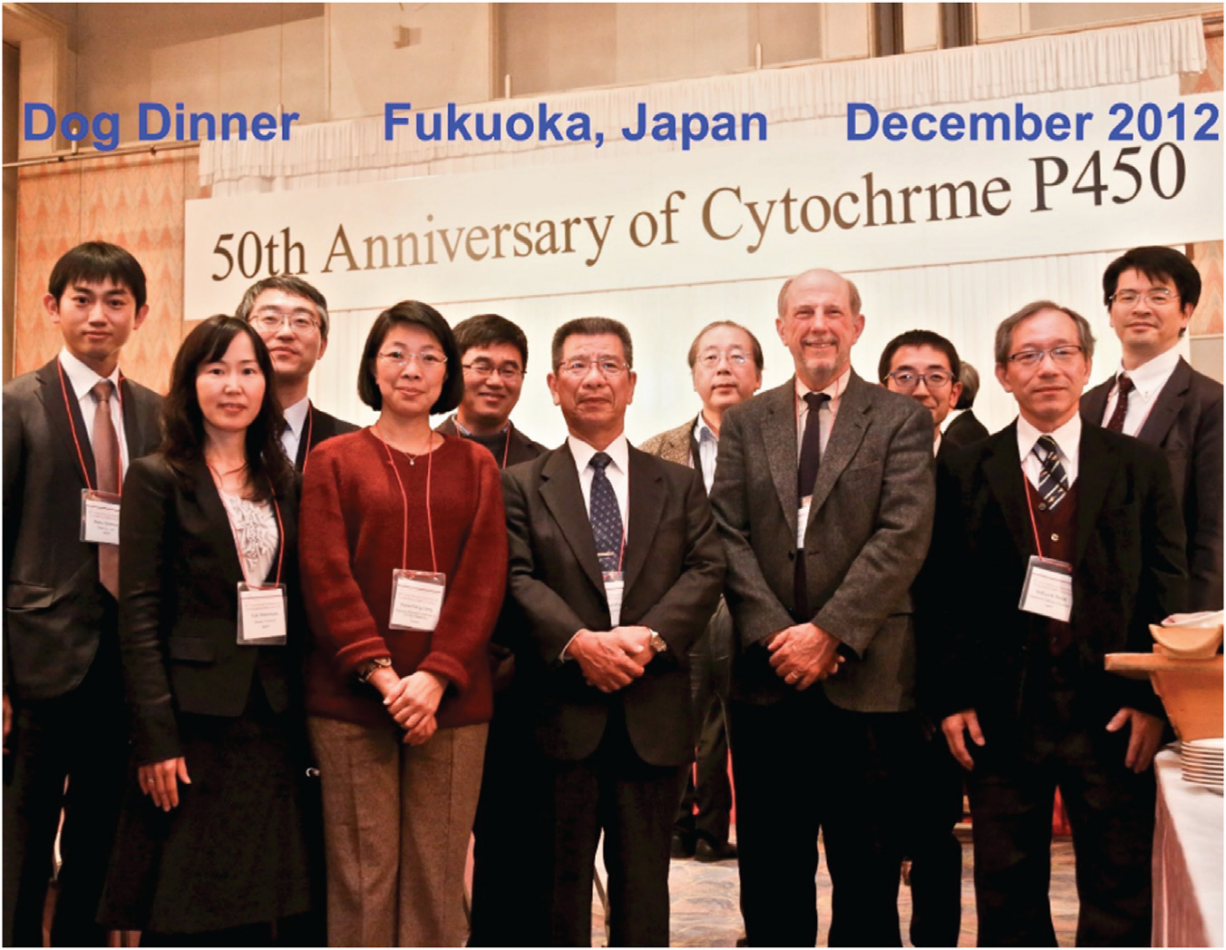
**Some former postdocs and students at a meeting in Fukuoka, Japan in December 2012 celebrating the 50th anniversary of the discovery of P450** (128) **and honoring Prof. Tsuneo Omura (note the unfortunate deletion of the “o” in “Cytochrome”).***Left to right*: Raku Shinkyo, Yuki Nishimura, Hideaki Sato, Yume-Fang Ueng, Chul-Ho Yun, Shin’ichi Yoshihara, Masahiko Iwasaki, the author, Kengo Watanabe, Nobuyuki Koga, and Hiroshi Yamazaki.

**Figure 9:**
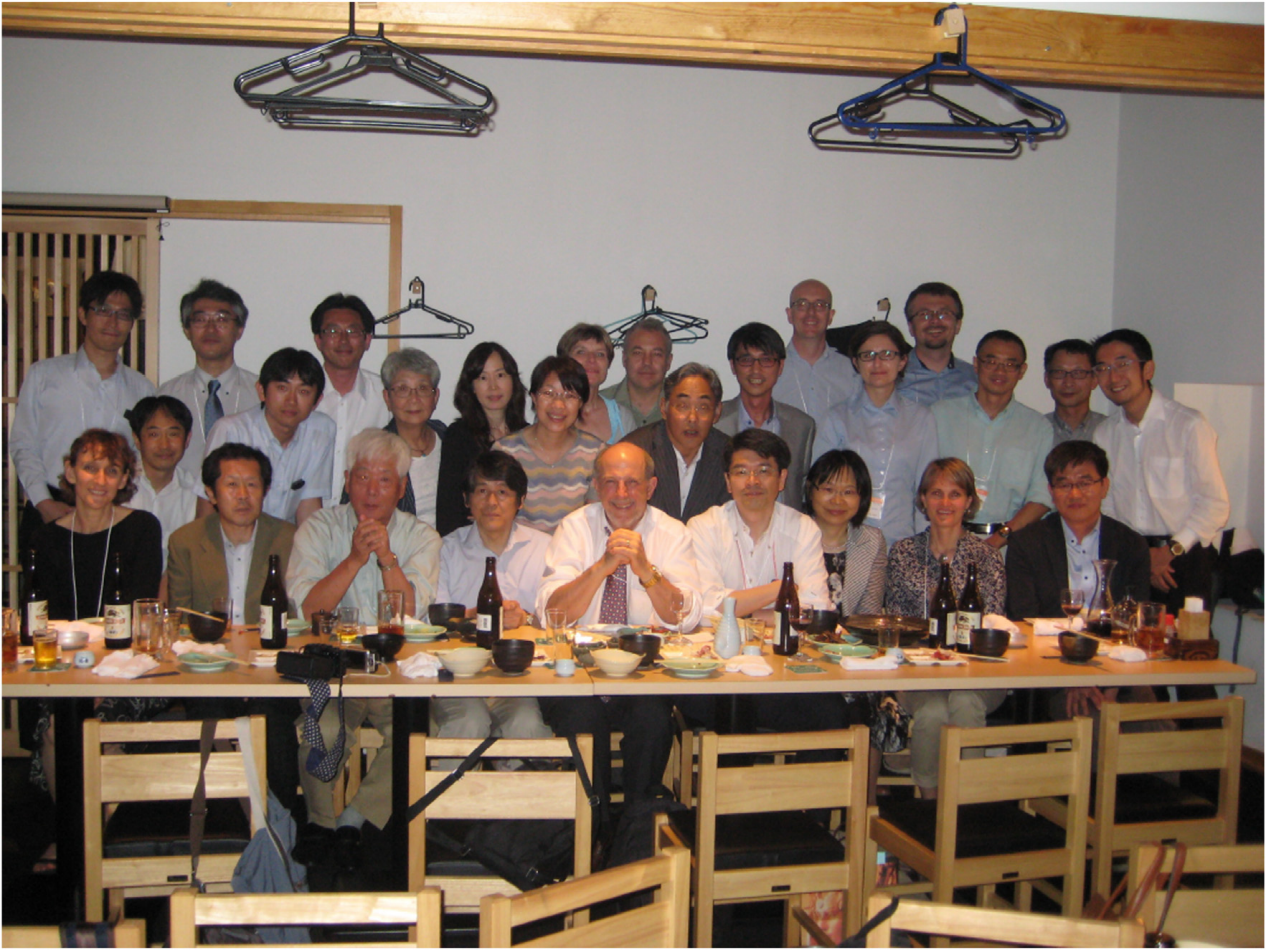
**Former graduate students, postdocs, etc. at a Dog Dinner at the 2015 ICCP450 meeting in Tokyo.***Front row, left to right*: Liz Gillam, Yasuo Seto, Tsutomu Shimada, Osamu Okazaki, the author, Hiroshi Yamazaki, Yukari Yamazaki, Laura Furge, Chul-Ho Yun. The Guengerich lab has the nickname of the “Dogs,” going back to the late 1980s. Reunion dinners are often held at scientific conferences and referred to as “Dog Dinners.”

**Figure 10:**
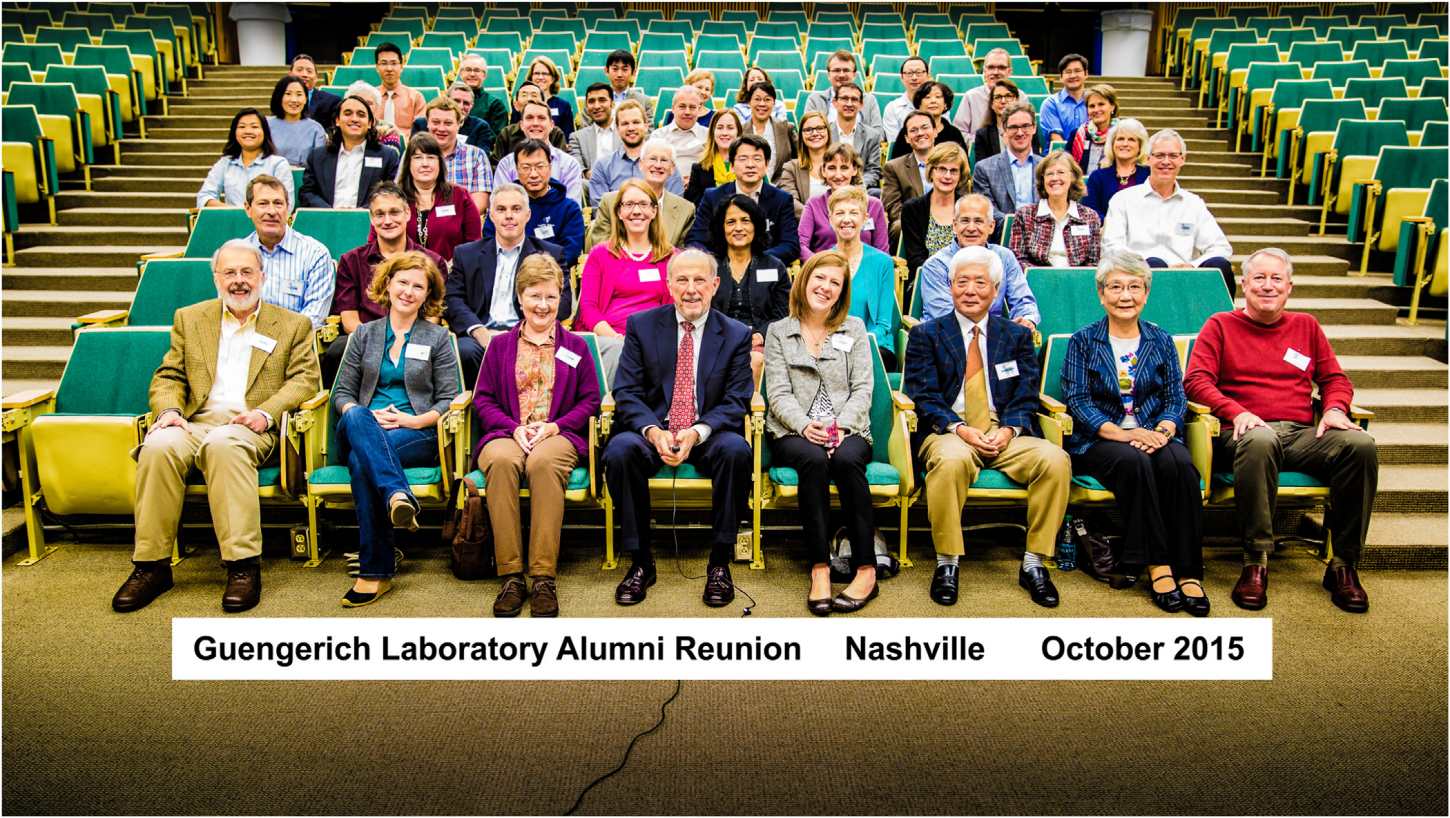
**Participants in the Guengerich lab reunion celebration at Vanderbilt (October 2015).** Attendees came to Nashville from 7 countries over 4 continents. *Front row*: Tom Harris, Anna Guengerich (daughter), Cheryl Guengerich (wife), the author, Laura Guengerich (daughter), Tsutomu and Setsuno Shimada, and Dan Liebler.

**Figure 11:**
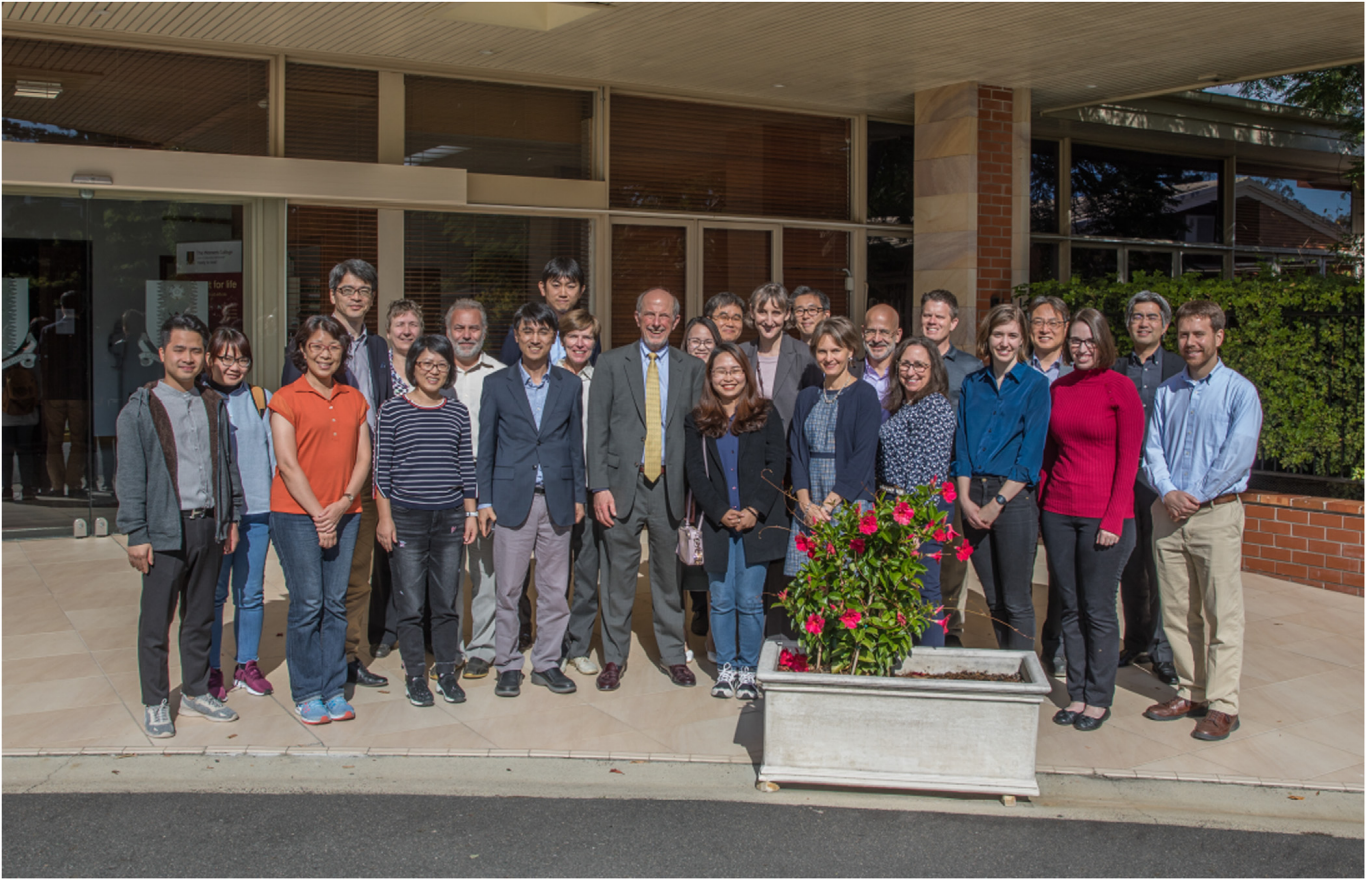
**A group meal (“Dog Dinner”) and celebration with some current and previous graduate students and postdocs.This wasat the 21st ICCP450 meeting (Brisbane, Queensland, Australia, 2019)**.

**Figure 12:**
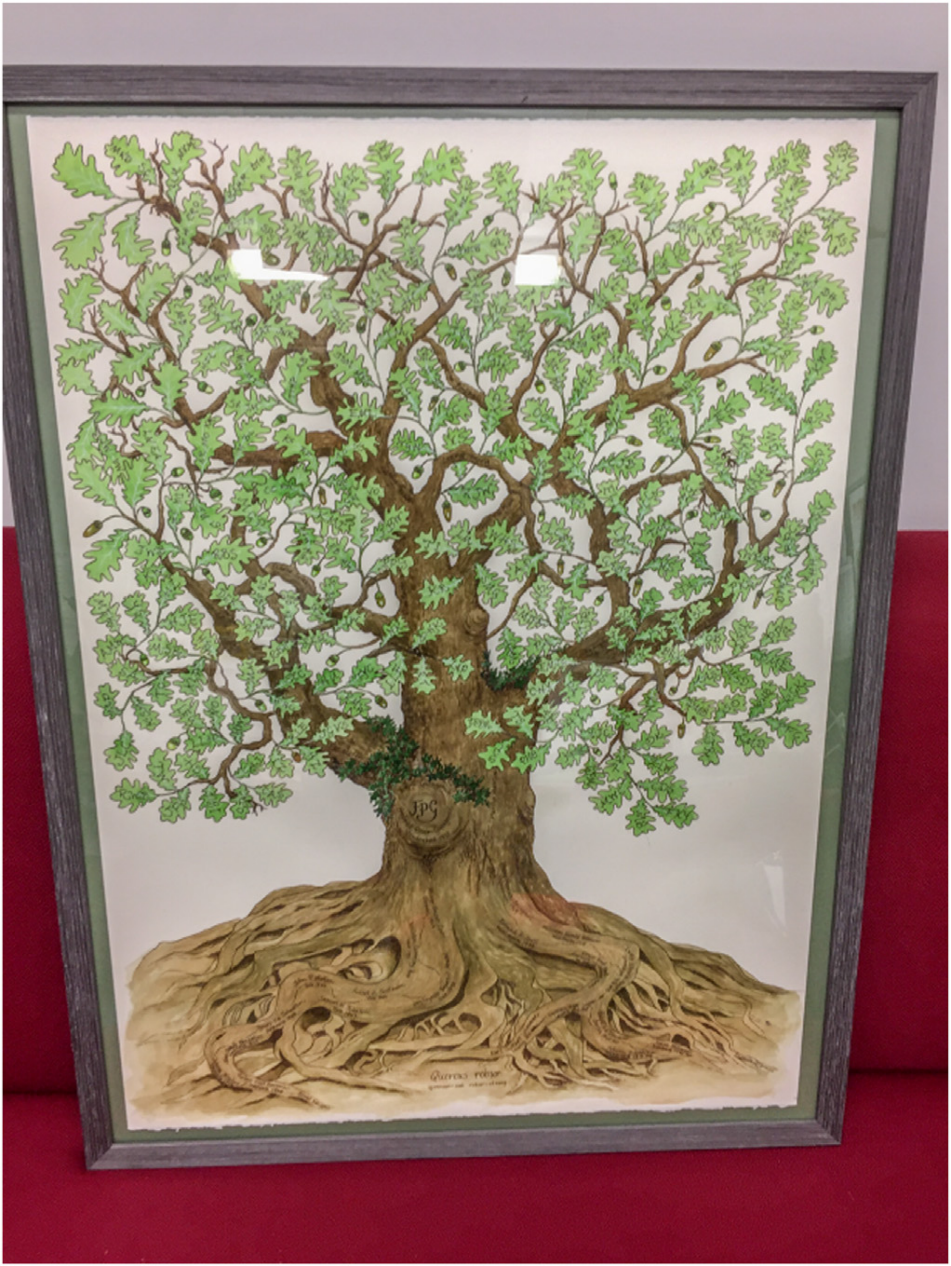
**A picture of a tree depicting my role (FPG initials on trunk), growing out of the roots of my mentors and their mentors** (101) **and generating the branches and leaves of my students and postdocs, with their initials.** Presented at the 2019 ICCP450 meeting in Brisbane, Australia and now hanging in the author’s office. Thanks again to Liz Gillam for the concept.

I have been approached seriously twice about leaving to take senior positions in pharmaceutical companies. These were attractive, in terms of the potential, and I probably could have had considerable influence. Ultimately, I turned these down, as I decided that I like working on basic problems with young people and mentoring them. Those relationships are priceless—see Figure 8, Figure 9, Figure 10, Figure 11, Figure 12. I guess I have not had any regrets (except when grant proposals did not make the cut).

In retrospect, I suppose I could have done more to build up Toxicology at Vanderbilt. Perhaps I was too selfish about my own research time, but it was important for my own students and postdocs. One issue was that the faculty in the Center in Molecular Toxicology were very good basic scientists, but perhaps we should have recruited new faculty with more interests in basic and applied toxicology. However, I had to run a program (Toxicology) with only the resources I could acquire myself—no institutional commitments of faculty positions, no space, and no money. I had to be very opportunistic but that can only take you so far. Nevertheless, the NIEHS-supported Center in Molecular Toxicology lasted about 48 years (56). Earlier, I had seen the area of nutrition in its zenith at Vanderbilt, and it too had faded. Other examples exist. There are life cycles in science. Nevertheless, I still consider toxicology to be very important, an area in which there are seldom any easy answers but a constant need for intelligent decisions.

Freed from the yoke of many administrative responsibilities, I continued to travel without feeling any remorse (Figs. 8 and 9)—well, I am not sure I really had any before.

Mike Waterman hung around Vanderbilt a couple more years (until retiring in 2014) but over time I inherited some of his P450 steroid projects, with P450s 17A1 and 21A2, both of which yielded X-ray structures with our colleague Martin Egli (75, 76, 77). Steroid metabolism presented several interesting scientific challenges that excited me. We also became interested in reaction processivity issues with P450s 17A1, 11B2 (78, 79), and 11A1 and 51A1 more recently (67, 74). P450 19A1, the steroid aromatase, was especially interesting and challenging. My student Christal Sohl devised a new expression and purification procedure, showing that the enzyme performs a 3-step reaction in a distributive manner (80). In what I consider a tour de force, we were able to revise the catalytic mechanisms of P450s 19A1 and 51A1, using ^18^O labeling and high resolution mass spectrometry on the third step (catalysis of C-C bond cleavage) and define the roles of compound I (FeO^3+^) and compound 0 (FeO_2_¯) (81, 82).

Since ∼ 2005, we have been chasing human P450 “orphans,” that is, P450s that did not have a home in the substrate categories (Table 1). We were successful in applying mass spectrometry–based metabolomic approaches to several of these (83, 84, 85, 86, 87, 88, 89, 90, 91). The function of P450 27C1 had been rather intractable (92). Its closest relatives (P450s 27A1, 27B1) use vitamin D_3_ as a substrate (Table 1). One day, I got a call from Prof. Joe Corbo at Washington University about his work with zebrafish, in which RNA-seq methods pegged all-*trans*-retinol as a substrate for 27C1 in fish eyes. This allows fish to change the spectrum of what they see when they migrate from salt to fresh water. We already had purified human P450 27C1, and then we found that it was a great catalyst for the 3,4-desaturation of retinol (vitamin A), retinaldehyde, and retinoic acid (93, 94, 95). We know a lot about the human enzyme, which is rather specifically expressed in skin (95, 96)—not the eye—but to date, we do not know exactly why so much of the retinoid pool in human skin is desaturated (1/4) (97, 98, 99).

**Table 1:** Classes of substrates of human P450 enzymes

Steroids	Xenobiotics	Fatty acids	Eicosanoids	Vitamins	Unknown
1B1^a^	1A1^a^	2J2	2U1^a^	2R1^a^	2A7
7A1^a^	1A2^a^	4A11	4F2	24A1^b^	2S1
7B1	2A6^a^	4A22	4F3	26A1	4X1
8B1^a^	2A13^a^	4B1^b^	4F8	26B1	20A1
11A1^a^	2B6^a^	4F11	5A1	26C1	
11B1^a^	2C8^a^	4F12	8A1^a^	27B1	
11B2^a^	2C9^a^	4F22		27C1	
17A1^a^	2C18	4V2			
19A1^a^	2C19^a^	4Z1			
21A2^a^	2D6^a^				
27A1	2E1^a^				
39A1	2F1				
46A1^a^	2W1				
51A1^a^	3A4^a^				
	3A5^a^				
	3A7^a^				
	3A43				

This classification is somewhat arbitrary in some cases, for example, P450s 1B1 and 27A1 could be grouped in either of two different categories, and some P450s have only very low rates of oxidation of fatty acids and derivatives.

a Crystal structure(s) available.

b Crystal structure of animal ortholog available.

## Celebrations

In October 2015, a group of my former trainees (headed by Dan Liebler, Griff Humphreys, Laura Furge, and Liz Gillam) organized a reunion symposium at Vanderbilt (Fig. 10). Former trainees from four continents attended—the people in Figure 10 are only a fraction of the 22 graduate students, 141 postdocs/visiting scientists, and numerous research assistants who have trained here. Dan said they were concerned that many of my trainees were already retiring, and they might not be able to make it if they waited until my own retirement. The individuals who attended the symposium spoke about their own career paths and what they had learned in my group that helped them. I was really touched by this effort. I have to admit that I could not see them all that well– the next month I had cataract surgery on both eyes. Enough about health—I cannot complain.

At the 2019 ICCP450 symposium in Brisbane, Australia, Liz Gillam organized a special session in the honor of my 70th birthday and several eminent researchers in the P450 field spoke about me and my contributions (Fig. 11). I also lectured on our recent work on the kinetics of substrate binding to P450s and the discrimination between conformational selection and induced fit mechanisms (66, 100). I was also touched by this effort. I have chaired the ICCP450 International Advisory Committee since 1997, appointed at the behest of Jud Coon, and it is a premier biennial international symposium in this field (and this year, I turned the job over to Prof. Emily Scott). Liz had also arranged an artist to make a figure of a tree with my scientific mentors and each of their mentors in the roots (101) and my own trainees in the branches and leaves (Fig. 12). This hangs in my office.

## Related disciplines

I have been privileged to be in biochemistry departments since I started graduate school. I suppose I could have worked in a chemistry or pharmacology department, but I still prefer biochemistry (and I am not a fan of having secondary appointments—why would I want to go to even more faculty meetings?). With my interest in drugs, the pharmacologists have been kind to me. Like ASBMB, ASPET (American Society for Pharmacology and Experimental Therapeutics) is a very good organization and it has given me some major awards (J. J. Abel (1984) and B. B. Brodie (1992) awards) even though I have never taken a formal class in pharmacology. I have also been active in the American Chemical Society (ACS), especially the Division of Chemical Toxicology, and still favor the chemical side of biochemistry. I was named in the first classes of Fellows in ACS and ASPET, as well as ASBMB. I have also been involved in the Society of Toxicology, International Society for the Study of Xenobiotics, and American Association for Cancer Research over the years. As I tell young people, train in biochemistry and then you can understand everything else (but know your chemistry).

As I mentioned, I chaired the International Advisory Committee for the ICCP450 meetings since 1997. These (biennial) meetings are great, but keeping free-standing meetings going is a challenge. It is always great to wrap up another successful meeting. I feel that I have helped accomplish something.

Over the years, I have been involved in outside consulting, mainly in the pharmaceutical industry. I learned a lot about how the scientific world outside a university really works. Occasionally, I have wondered if I was neglecting my lab members while I was getting paid for doing this, but my contacts in industry did help many of my people get good jobs. It was interesting to be paid to do things that I was expected to do for nothing most of the time at home. Beginning in 1994, I also got involved in consulting with law firms sometimes, mainly involving drug patent cases. I have found many of the individual lawyers very bright and knowledgeable in both law and science. I also learned from them how to argue and to look at issues from both sides to spot the weak points—also, not to say more than you can readily defend. Good lessons. I still do some of this, although the cyclical nature of the work can be frustrating.

Since 2015, I have served on the expert panel of the Flavor and Extract Manufacturers’ Association, which reviews and approves flavor additives to food. I have enjoyed using my expertise in analytical chemistry and toxicology to make decisions about whether to approve the uses of chemicals in food for the FDA. I guess I have come full circle since starting college in food science (*vide supra*), but in a different way.

## Things we had to wait for in terms of technology and new insights

Sometimes questions are very difficult to answer unambiguously without the right technology or a key new reagent/approach, and you have to be patient. A prime example is our work on the oxidation of trichloroethylene (TCE) and the reactions of TCE epoxide. My first graduate student, Randy Miller, did a really admirable job on TCE (22, 102) but it was not until LC-MS was developed a decade later that my postdoc Hongliang Cai was able to complete it (62). Several questions about P450 kinetics, particularly burst kinetics in product formation, were unresolved until we got our rapid-quench apparatus in 1994 and my student Chenie Bell went to work with it (103, 104). Although we had considerable evidence that P450 FeO complexes could do stepwise electron transfer with amines (44, 45, 63, 105, 106), later work with steady-state tetramethoxybenzene oxidation was unambiguous (64). Gel electrophoresis results on DNA polymerase–mediated misincorporation were complex and confusing until my postdoc Hong Zang developed a nice LC-MS approach to sequencing the products that I already mentioned (70). Our early efforts to crystallize a DNA polymerase in the act of making a misincorporation opposite a DNA adduct were very unsuccessful with HIV-1 reverse transcriptase but quickly succeeded when we switched to Dpo4 (and collaborated with Martin Egli) (70). For many years, we were interested in measuring the interaction of cytochrome *b*_5_ with P450s but were never successful until a serendipitous tour of fluorescent dyes led us to labeling with Alexa Fluor 488 (107).

Over the years, we have been interested in the more unusual reactions that P450s catalyze, and I have written some reviews on this topic (108, 109, 110, 111). Almost all of the oxidations can be rationalized in the context of compound I (FeO^3+^) or, in a few cases, compound 0 (FeO_2_¯) chemistry (82, 112). Two non-redox P450 reactions are still a mystery to me (113, 114).

I can also mention projects that were never successfully completed. One of the goals Harry Broquist had for me as a graduate student was to define how pipecolic acid was converted to an indolizidine ring in alkaloids (1). I never got around to this and eventually (50 years later!) the biosynthesis appears to involve a hybrid nonribosomal peptide-polyketide synthase (115), which we never envisioned (not even sure these were even known then). As a student and assistant professor, I could not really identify exactly how the alkaloid slaframine is activated to its pharmacologically active form (116, 117). Although we discovered and published extensively on the DNA adduct *S*-[(*N*^7^-guanyl)ethyl]glutathione (formed from ethylene dibromide), we could only provide indirect evidence, but not proof, that it is directly miscoding (118).

We are still struggling with some “orphan” human P450s (Table 1)—we still have no substrates for P450 20A1 (119). While P450 27C1 is clearly a retinoid desaturase in skin (95), we do not know why human skin contains a high fraction of 3,4-unsaturated retinoids (99). Perhaps some of these questions will ultimately be resolved.

## Some things get noticed and others do not

Several of the research projects we have published on have garnered a lot of interest—and citations (*e.g.*, Clarivate or Google). In particular, a number of our studies on P450, particularly human P450s, have been highly cited. The most highly cited paper I have published, on the immunochemical quantification of individual human P450s (120), was not particularly mechanistic and the technology of proteomic approaches is probably superior today—but it keeps getting cited (currently ≥ 3500 Google citations). What is surprising is that some of the detailed mechanistic chemical and biochemical studies—which often required considerable time and intellectual energy—never did get cited all that much, for example (62, 70, 81, 121, 122, 123), nor did our evidence against a major *in vivo* role of P450s in generating reactive oxygen species (124, 125).

## Things I got unexpectedly and things I did not get done

As I look back on my career in biochemistry, there were some awards and positions I received that I had not expected: First of all, I had not expected to be the Director of the Center in (Molecular) Toxicology—I disdained administrative work when I started on the faculty, but this fell to me in 1980/1981. I had not really expected to ever receive the Sutherland Prize at Vanderbilt (*vide supra*). I had not expected to be an AE at JBC, but this happened and lasted for 16 years. I had not expected to receive ASPET’s B. B. Brodie Award (in Drug Metabolism)—a lifetime achievement award—at the age of 43. None of these organizations elected Fellows until recently, but I was in the inaugural classes for ACS, ASPET, and ASBMB. Finally, I never expected our work to be as highly cited as it is.

I had not expected to get an endowed chair in my department (I always thought the administration assumed I would not leave anyway).

There were some awards that did elude me. I applied to be an American Cancer Society Professor twice unsuccessfully (when the cancer community still cared about our work). Although nominated several times, I was never elected to the National Academy of Sciences or the National Academy of Medicine. I doubt if I will ever receive any more awards. That is okay. In retrospect, none of these shortcomings bother me—I would probably not have been any happier than I am now. At this point in my life, I know who I am, and I do not have to prove anything.

## Today

Fast forward to late 2023, the time of writing—I am still in business (pending NIH decisions!). One of the concerns about writing a Reflection is that people might think I am retiring and will not be accepting new student and postdocs. That is not the case, at least not yet. After ≥765 primary papers, 322 invited reviews, 138 book chapters/meeting reports, 22 graduate students, and 141 postdocs/visiting scientists, I have still not lost any of the excitement about biochemistry that I learned from Harry Broquist in 1968. I was blessed to have two excellent mentors in Harry Broquist and Jud Coon, and I have tried to be a good one myself. Maybe I have—at least I received four mentoring awards (plus the ASBMB Rose Award), and the Vanderbilt award for mentoring postdocs in research is now named the Guengerich Award. The awards are not the real reward—in the last analysis, working with and training young people has been as important to me as the research itself (Figure 8, Figure 9, Figure 10, Figure 11, Figure 12). I like to think that I have had some positive influence on many young scientists.

I am somewhat unusual in that I have always worked in the lab myself, at least to some extent, even when I had more administrative and editorial responsibilities. I cannot perform some of the newer techniques used in our lab, but I am still fairly good at organic synthesis, spectroscopy, and kinetics. Perhaps I am trying to emulate Vince Massey (*vide supra*). I still relish the technical accomplishments, and last year (2022), I successfully completed a 17-step organic synthesis myself to make some labeled sterol substrates we needed (67, 82).

I started out as just a “kid from the farm” (126). After my parents died, I bought my sisters’ shares of the farm back in Illinois and I manage that, working with tenants Karl and Dale Glueck. We raise only corn and soybeans. I basically farm by phone, but I do have to make decisions about storing and selling the grain, so I follow the market trends weekly and keep abreast of developments in agriculture. As I think back on my life, I learned a lot from my father and the other farmers. Not only did they work hard—they were very resourceful and self-reliant, as well as honest. Those qualities are hard to learn and teach.

**Figure 13:**
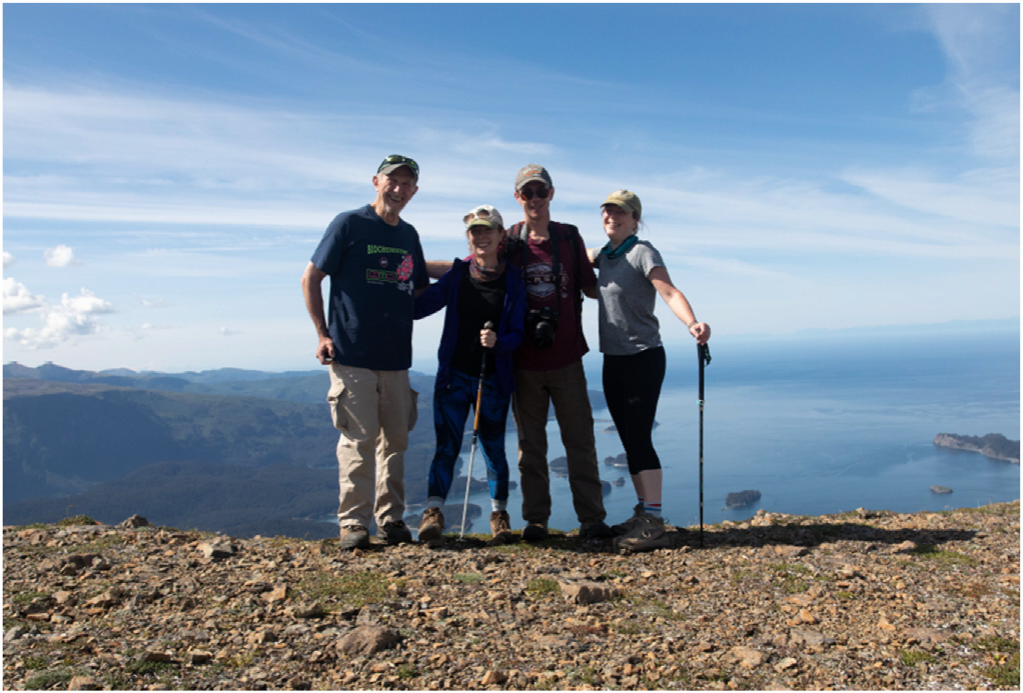
**The author (*left*) and his three children (*left to right*: Anna, Phillip, Laura).The photo was taken (by author) after a hike to the summit of the Grace Ridge Trail in Kachemak Bay State Park, Alaska in 2020 (1000 m elevation)**.

**Figure 14:**
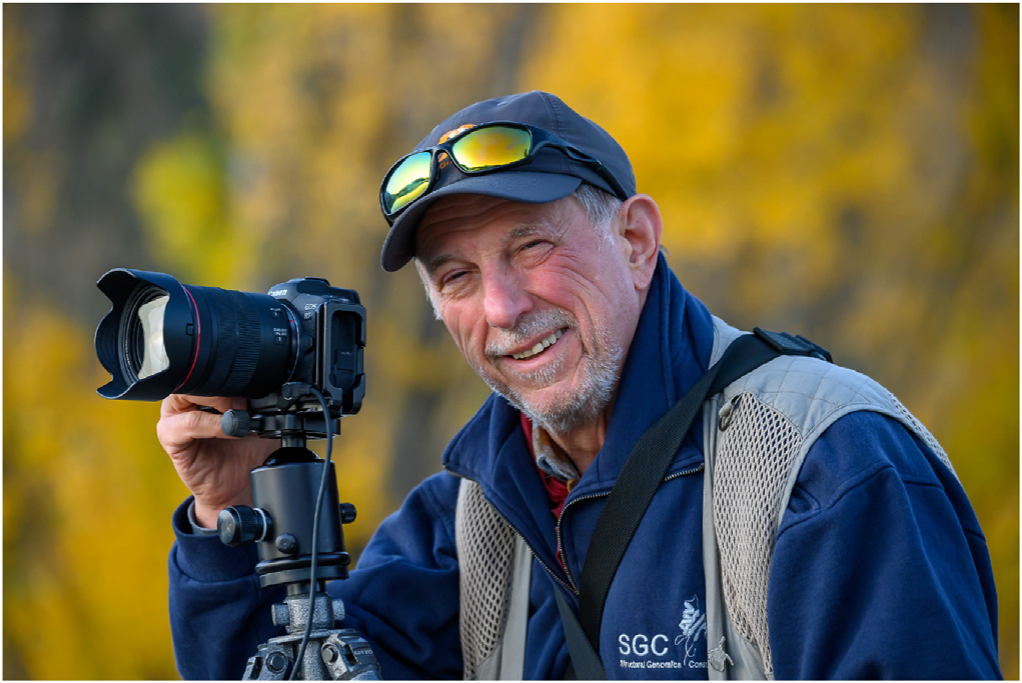
**The author working with some of his equipment. This photography was takenin New Zealand (April 2023)**.

As time went by, I eventually developed two hobbies, fishing and photography. My wife Cheryl and I have been vacationing for a couple of weeks in Alaska every year since 2004, where I spend about half my time fishing for salmon and halibut and also doing some hiking (Fig. 13). I have learned more about photography and take expedition trips each year with Muench Workshops. Last December, I went to Antarctica, an absolutely beautiful place, and Patagonia—then later to New Zealand (Fig. 14). Work hard; play hard.

**Figure 15:**
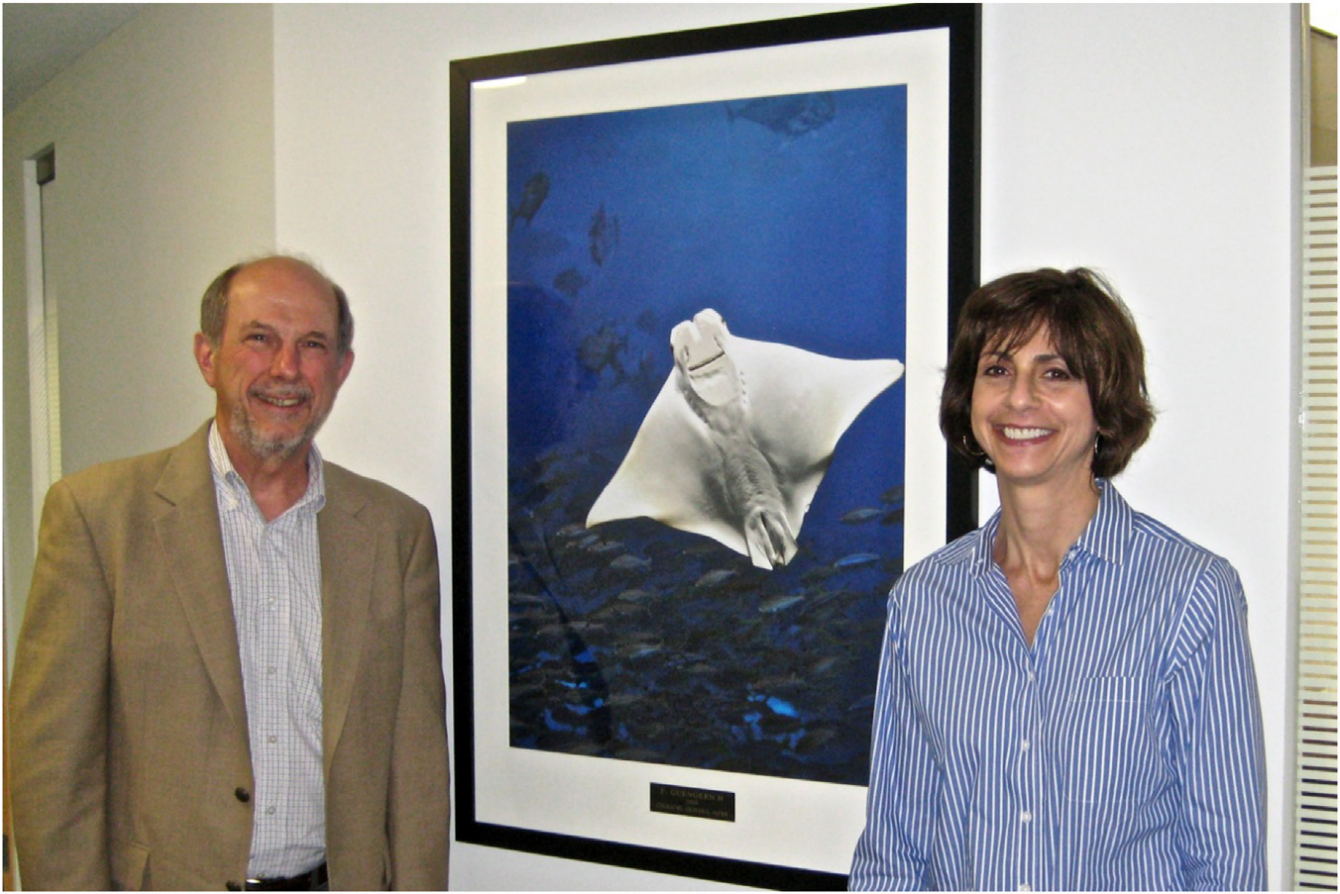
**At the ASBMB office with one of the author’s pictures (taken in Okinawa at the ICCP450 2009 meeting).** The author and Nancy Rodnan.

JBC is an ASBMB journal, and I should mention that (thanks to Nancy Rodnan and some individuals formerly at ASBMB) several of my photographs are framed (24 × 36 inches) and grace the halls of the ASBMB office (Fig. 15). I hope that these have given the staff and visitors some pleasure; it is my privilege just to share them.

## Final lessons and advice

I think I could have been happy, based on my agrarian youth, being a cattle breeder or running a large farm (*vide supra*). However, I would have missed out on many things I never knew about when I was young. I have been able to travel to more than 40 countries over all 7 continents. Moreover, I met many people in my career, who taught me that people are really basically the same everywhere and that there is only one race, the human race. Ethnicity is real—and interesting—but people are people and should be treated that way. I have been able to gain insight into how individuals in different places live and what drives them. As I have already mentioned, I have been able to have some influence into how many of the people in my group developed their careers. Most of the research questions I addressed would have probably been answered eventually without me anyway, so perhaps my mentoring career has been even more important.

I learned from my mentors— both what to do and what not to do. You cannot totally pattern your life after that of someone else because you are not in the same situations. I could not get through my own problems by thinking “what would the Chief (Harry Broquist) do” or “what would Jud (Coon) do?” Besides, sometimes they did the wrong things, in retrospect (so have I). Ultimately, it is your life and you have to shape it yourself.

Working hard was something I learned from my father. Alongside Harry Broquist and Jud Coon, he was a major influence in my life. I did not take that much time off when I was younger (and I also had family responsibilities when our children were young).

Treat people fairly. This has been very important as an editor and also in dealing with students and postdocs. Many of my trainees became life-long friends (Figure 8, Figure 9, Figure 10, Figure 11).

I have found biochemistry and chemistry to be a very interesting career. I think it is fine to have programs to interest young people in “STEM” careers (science, technology, engineering, mathematics) but this business is more than “gee whiz” work. I have seen many cases of individuals who did well in classes, simple lab exercises, and (even in grad school) rotation projects but never got beyond that. The people who were successful were those who solved difficult problems because they thought about what they did, interrogated every little step to find out why things were not working, and persevered. It is not always easy to identify (and recruit) these people (this is advice to young faculty members picking students). Most of your experiments fail, and you need the fortitude to identify the issues and solve the problems.

There are many things that you are not taught in graduate school. Most have to do with handling people, and the cases are often unique. One of the more complex problems I had was with my former postdoc, long-term collaborator, and friend Dr. Tsutomu Shimada. He made many short-term visits to our lab over the years and was extremely productive, in that he knew what he wanted to do and where everything that he needed in my lab was. People in my group were amazed (dismayed?) that he would be often getting results by the second day after he arrived and then organizing a manuscript by the end of the first week—great. However, there is (was?) a tax agreement between the United States and Japan in that Japanese visitors are exempt from U.S. taxes for the first 2 years. However, every time Tsutomu arrived, our Human Resources people at Vanderbilt assumed he was new and did not take taxes out of his salary. Tsutomu kept a bank account in Nashville for convenience, in that he came so often. One day, he sent me a message from Japan saying that the bank balance was now zero and the Internal Revenue Service (IRS) had a lien on his account —and asked “what is a ‘lien’?”. Wow. Well, the IRS said he owed $25,000 in taxes, penalties, and interest. Tsutomu could have ignored this but would have been arrested if he tried to enter the U.S. again. Finding an accountant or lawyer in Japan who understood our IRS would probably cost that much (it is hard enough for us in the U.S. to understand the IRS), so I agreed to help him. After numerous exchanges with the IRS over the course of a year (and a myriad of forms), I finally got them to drop the penalties and interest due to his ignorance of the tax laws, so he only had to pay the basic taxes. This was not fun, but I have more papers co-authored with Tsutomu Shimada than anyone else—I guess I owed him something.

Again, you cannot really model your career on that of your mentor(s). As far as I know, neither the Chief nor Jud had to do tax accounting for trainees. Do watch your mentors and note what they do well—and what they do not do well. The same applies to watching me.

I have a number of thoughts about teaching and the positive and negative changes over the years. I still teach graduate courses and enjoy it. Jud Coon told me that (in his experience) “everyone wants the title of professor but no one wants to teach anything.” I see his point. I do not have the space to elaborate on exactly what all my thoughts are about teaching, but I do have a lot of ideas. Last year, I presented a number of “Master Class” lectures at the University of Queensland (Brisbane, Australia) at Liz Gillam’s invitation. There were very well-attended and I was gratified about the interest people showed there.

Be honest and open-minded. As I got involved in some patent consulting work with lawyers, I learned that they looked at their cases from both sides to anticipate arguments. When you write your papers, think hard about what the deficiencies are and what you could criticize as a reviewer.

Details are important. One potential danger about having a larger laboratory is that these are more likely to be overlooked. Do not assume that everything has been done properly. Has your student done every necessary control experiment to establish the conclusions? The issue extends beyond the lab, and you may overlook even what some of the staff and others neglect to do, even if they are good most of the time. Are the budget reports you have been getting from the accountants really accurate? Is the computer backup system your tech person set up really working? Did the travel agent neglect to tell you that you need a visa for a foreign trip? I have been stung on these things. Always check on everything.

If you have a family, they are important. I have had a supportive wife, Cheryl, for nearly as long as I’ve had my degree (50+ years). I love our three children very much (Fig. 13). They mean a lot to Cheryl and me.

Take care of your health. This job requires stamina.

Do not waste money. Getting resources is hard in academic life. In general, I was only able to acquire major equipment with money I had left over from the personnel budgets. Take good care of the equipment in your lab, because acquiring it is usually not easy in an academic lab setting. It is hard to get the money, so make the equipment last. I still use two preparative centrifuges I bought when I started in 1975. Be sure that the people in your lab share this attitude—or else change that behavior!

Keep active in the lab as long as you comfortably can. It will give you a better sense of judging how hard specific problems really are. Some of your trainees will see you as a good model (but not all will care). You will also better understand what shape equipment is really in. Two good examples of scientists who continued doing lab work for a long time were Vince Massey and Stan Cohen, both already mentioned.

Not all collaborations work out. Some efforts have been great but others fizzled. Be careful in getting into these, especially when you are starting out. You cannot waste your time.

Learn to write well. In some ways, this was hard for me. I have already mentioned that as a young man, I was only interested in things related to chemistry and math. I disliked studying English, and as I said, I had very limited coursework in foreign languages. Today, I spend much of my time writing or criticizing what others write (especially when I was an editor). Jud Coon was a real stickler about writing. He gave me back drafts with marks and changes all over. I thought “Doesn’t he like *anything* I write?” I think I learned the importance of writing though. (By the way, you cannot write well if you do not read a lot.) Later in my career, Jud told me he thought my own papers were well-written—and eventually, I became an editor and critiqued others. Then I really appreciated good writers.

I have mellowed somewhat from my early days. That is probably not unusual. I have also learned that arrogance is something to avoid (not convinced everyone learns that). Be careful in hyping yourself. You *could* be wrong. If your work is important, others will (probably) let you know. At least treat those you disagree with respectfully and do not get personal about your differences.

Also, have a sense of humility when dealing with non-scientists. I realized I was a true amateur in many areas when I took up exploits like photography and fishing. Other people are experts at things outside of science, and you are an amateur. Also (a lesson from the Covid-19 era), do not try to arrogantly impose ideas on others in the general vague name of “science,” especially if there are still real scientific disagreements—or a real lack of evidence. Remember how science works, with hypotheses to address, etc. There is a difference between science and scientism.

I have concerns about current aspects of our scientific enterprise, although I do not have space to cover all of them (and hate to end on an adversarial note). Although NIH has been my almost exclusive benefactor, I have serious concerns about recent trends there, aside from limited resources (have you heard the term “reputation bias” (127)?). I also have concerns about some of the directions I see in universities, in general. I am not convinced I would even be hired today. Finally, I have concerns about my own scientific field in general, in terms of what will be left and who will be solving the remaining questions—and who will be training young scientists.

In retrospect, I am unusual in many ways, even among other scientists. I am thankful that I have a life that has been so interesting. Biochemistry is great because it provides a background to apply to so many kinds of problems. In my case, it involved basic chemical mechanisms, how enzymes work, drug metabolism, toxicology, endocrinology, biosynthesis of natural products, analytical chemistry, and other things. I learned a lot about how the pharmaceutical industry works and the good and not so good things about government agencies. I have seen how lawyers work and been to court (representing others, not myself!). I have been involved as a consultant in an FDA hearing for a (successful) new drug. I understand how many drugs work and a lot about adverse drug reactions.

Despite some of my misgivings, things invariably change and if I have learned one thing, it is that you have to roll with the punches. You have to be able to adapt, at least in some things. The U.S. Navy SEAL motto is “The only easy day was yesterday.”

## Conflicts of interests

The author declares that he has no conflict of interest with the contents of this article.

## Footnotes

† This was when I first began using my name as “F. Peter Guengerich”—simple reason: there was a length limit for names on that plaque in the University Library and my full first name (Frederick) did not fit. I liked the ring of it and have used it much of the time since, except on my passport, driver’s license, etc. I also honor, in some way, my father and grandfather, both named Peter Guengerich. To most people I know, I go by “Fred.”

‡ I hate to bring this up, but this paper (16) was first submitted to JBC but declined because it was considered only a “methods” paper, which at the time was not desired at JBC (at least in principle, although there were some exceptions–or papers that simply sneaked through). So we then submitted the paper to Biochemistry, who were happy to publish it. Since then, the JBC has changed its policy and now seeks methods papers, which are sometimes cited much more, for example Lowry *et al.* (21). This paper of ours has now been cited at least 521 times (Clarivate) but at this point, few scientists if any think of it when running “Western” blots, quantifying, etc.
